# Acoustic micronektonic distribution and density is structured by macroscale oceanographic processes across 17–48° N latitudes in the North Atlantic Ocean

**DOI:** 10.1038/s41598-023-30653-5

**Published:** 2023-03-21

**Authors:** Eva García-Seoane, Thor Klevjer, Kjell Arne Mork, Mette Dalgaard Agersted, Gavin J. Macaulay, Webjørn Melle

**Affiliations:** Institute of Marine Research (IMR), Nordnes, P. O. Box 1870, 5817 Bergen, Norway

**Keywords:** Ocean sciences, Marine biology, Physical oceanography

## Abstract

This study investigates the large-scale distribution patterns of the acoustic scattering layers and micronekton density across the Northeast Atlantic Ocean during daylight hours. A research cruise on board R/V “Kronprins Haakon” was conducted during May 2019 from Cape Verde to Bay of Biscay. Hydrological data were obtained at 20 conductivity-temperature-depth sensor (CTD) stations. To estimate the micronekton densities in front of the trawl, an autonomous echo sounder (120 or 200 kHz) on the headrope of the macroplankton trawl was used. Acoustic data were also collected along the cruise track using ship-mounted multi-frequency echo sounders (backscatter at 18 and 38 kHz was analyzed). Acoustic observations (both at 18, 38 and 120/200 kHz) showed clear patterns in the horizontal distribution of the micronekton during daytime with higher backscatter and echo densities in the south of the study area (from 17 to 37° N), and the absence of high backscatter in the surface from 37 to 45° N. Backscatter and echo densities were found to be significantly influenced by: temperature, salinity, and oxygen, as well as depth and time of the day.

## Introduction

Micronekton are major predators of zooplankton and are important prey for top predators (e.g., tuna, swordfish, turtles, seabirds and marine mammals)^1,2^. Many species of mesopelagic micronekton conduct diel vertical migrations (DVMs) from depth during the day to shallower waters at night and back to depth at dawn (e.g., Drazen et al.^3^). Through these DVMs, micronekton actively transport organic material, contributing to the biological pump^4^. Aggregations of micronekton and macrozooplankton in the mesopelagic zone comprise the deep scattering layers (DSLs), which are strong and ubiquitous sound-reflecting layers in the open ocean (e.g., Davison et al.^5^). There is an increasing interest in micronekton (especially mesopelagic fish) as a potential resource for commercial exploitation because of its high estimated biomass of fish^6^, but the current estimates of abundances and biomass have high uncertainties^7,8^.

The micronekton are small organisms (1–20 cm) that can swim, but most studies implicitly treat mesopelagic biota in the micronekton and macrozooplankton size range as functional plankton, i.e., not capable of undertaking horizontal migrations (but see for instance Benoit-Bird and Au^9^ for a possible exception). Describing large-scale patterns and explaining the processes that drive them thereby becomes relevant. For example, understanding how latitudinal gradients in environmental variables influence the distribution and abundance of the organisms may be useful for predicting the effects of climate change on ecosystems and their components^10^. Large scale studies provide a good opportunity to explore the effect of macroscale oceanography in the horizontal and vertical distribution of micronekton, and consequently the responses of the organisms of the DSL to environmental variability. It has been suggested that DSL depths are controlled by light^11–13^ and appear to follow specific light intensities^14^. However, DSL depths have also been linked to other environmental variables such as oxygen levels^15–17^.

In addition, biogeographic classification is an essential tool to reach international agreements in marine conservation^10^. In the past, biogeographic partitioning of the ocean was in general conducted with just biological data, but more recent classifications including numerous data sources (e.g., biological, chemical and physical) into their partitioning algorithms (e.g., Proud et al.^18^). Sutton et al.^19^ presented a global biogeographic classification of the mesopelagic zone in terms of biodiversity and function. They define a total of 33 global mesopelagic ecoregions, and our study spanned 3 of them. On the other hand, Proud et al.^20^ defined 36 mesopelagic provinces based on the characteristics of observed acoustic backscatter distribution.

The main circulation patterns of the Northeastern Atlantic and how it affects the hydrographic conditions in the study area are detailed in^21^. Even though the Northeast Atlantic is one of the most studied areas (e.g., Magnússon^22^, Peña et al.^23^, Ariza et al.^24^, Blanluet et al.^25^, Cascão et al.^26^ there still are major gaps (e.g., accurate estimations of biomass and knowledge on interaction between oceanography and micronekton biodiversity and biomass are needed) in the biology and ecology of the micronekton^6^. The Northeastern Atlantic is a potential area for future fisheries, and commercial vessels have recently obtained preliminary licenses for experimental trial fisheries of micronekton^27^. Thus, it is important to improve our understanding of the overall structure and composition of the micronekton in this area. The objective of this work was to investigate large-scale distribution patterns of acoustic scattering layers and densities of micronektonic and macrozooplanktonic organisms (hereafter micronekton for simplicity) together with their oceanographic habitats in the Northeast Atlantic Ocean using acoustic techniques.

## Material and methods

**Figure 1:**
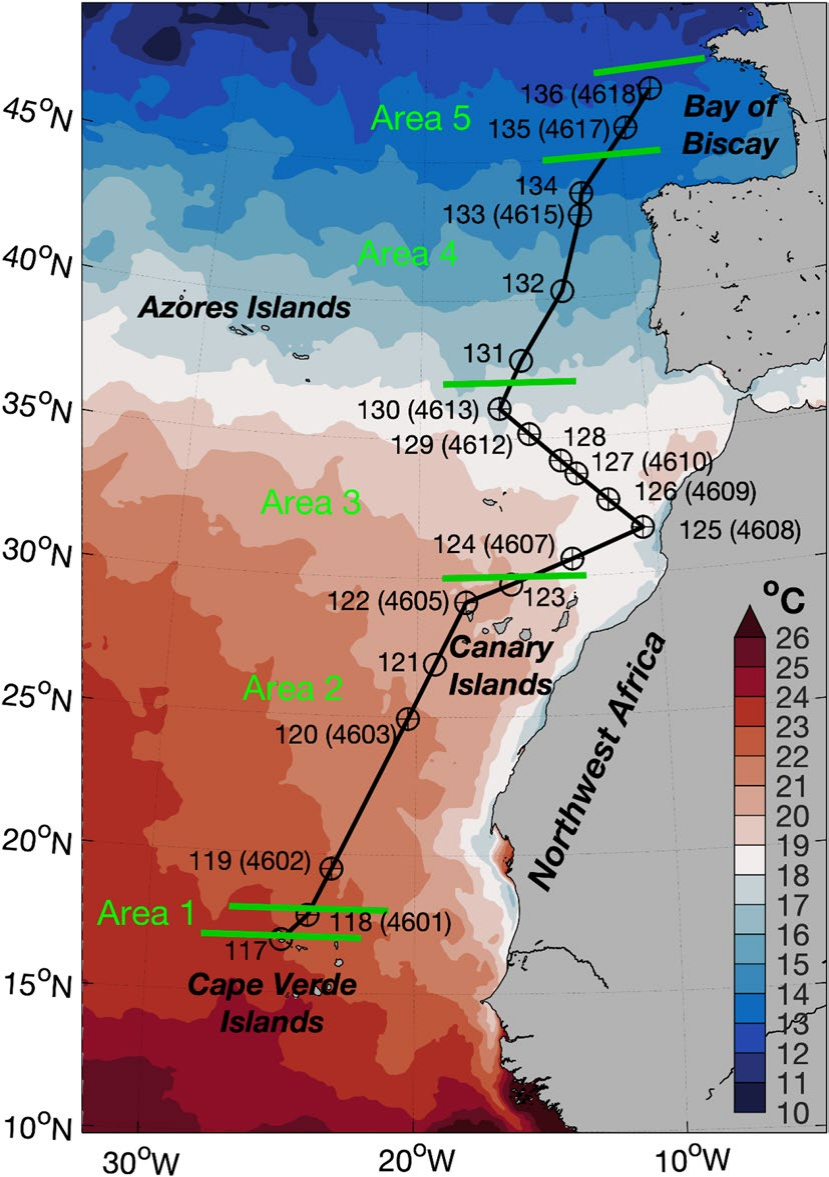
Map of the study area with sea surface temperature (°C) averaged over the period 2–22 May 2019. The Sea Surface Temperature (SST) data are daily means from satellite data and product of the Operational Sea Surface Temperature and Ice Analysis (OSTIA) system run by the UK Met Office^30^. Locations of the CTD stations (circles), and macroplankton trawls (+). The CTD stations are numbered from 117 to 136, and the trawl stations (in parentheses) from 4601 to 4618. Only stations used in the analysis are numbered. The green lines represent the five areas defined in this study according to the distribution patterns of acoustic backscatter observed (see “Results” section below). This figure was created using MATLAB, version R2018a from MathWorks (https://www.mathworks.com).

Data were collected during a research cruise onboard R/V *Kronprins Haakon* from 2 to 22 May 2019 in the eastern part of the North Atlantic Ocean from Cape Verde to southern part of France (17° N 25° W to 48° N 8° W) (Fig. 1). A total of 20 stations were sampled along the cruise transect mainly during daytime. A macroplankton trawl^28,29^ with a mouth opening of ~ 34 m^2^ and a mesh size of 8 mm stretched (squared meshes with 3 × 3 mm light opening) was towed obliquely at a speed of around two knots from the surface down to 1200 m depth and back to the surface at 15 stations. A total of 5 stations have not been analysed in this work because two of these stations consisted only of a CTD deployment, and the other 3 stations were sampled with a pelagic trawl without the WBAT attached. A Wideband Autonomous Transceiver (WBAT, Simrad) with a 120 or 200 kHz transducer pointing horizontally forward was attached to the headrope of the macroplankton trawl to acoustically count and measure the organisms in front of the trawl.

### Oceanographic data

At each station, a Conductivity, Temperature, Depth (CTD) profiler cast was conducted down to 1200 m depth using a SeaBird 911plus probe. The CTD also carried sensors for measuring dissolved oxygen concentration (SBE 43), and fluorescence (Wet Labs ECO-FL). The oxygen and fluorescence sensors were not calibrated, and those values are treated as relative.

**Figure 2:**
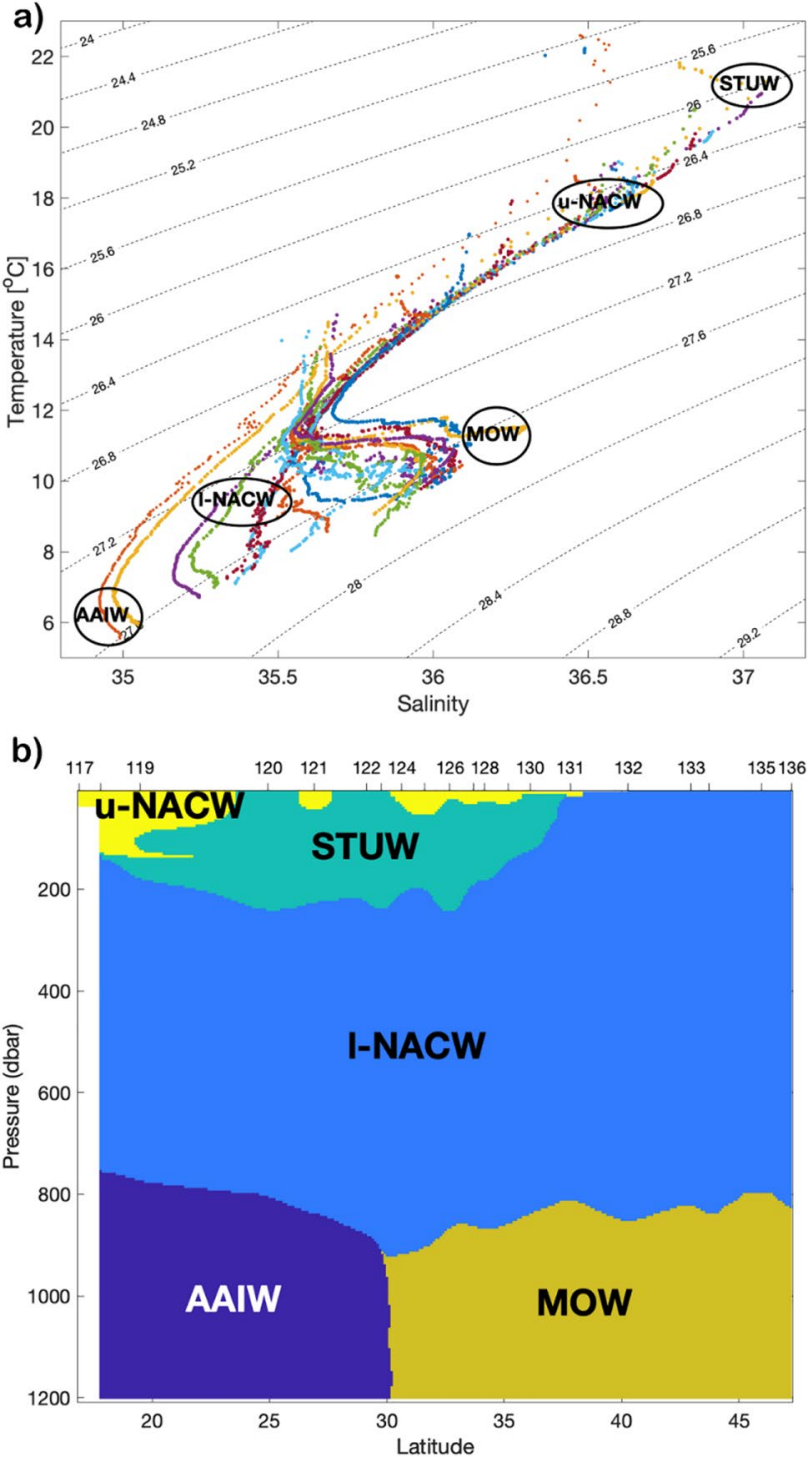
(**a**) Temperature-salinity diagram for all stations, each with different color, and schematic locations of water types: upper North Atlantic Central Water (u-NACW), lower North Atlantic Central Water (l-NACW), Subtropical underwater (STUW), Antarctic Intermediate Water (AAIW), and Mediterranean Overflow Water (MOW). (**b**) Overview of the different water types along the cruise track. The CTD stations (from 117 to 136) are indicated on the upper side of the plot. This figure was created using MATLAB, version R2018a from MathWorks (https://www.mathworks.com).

To investigate the role of water mass properties on the micronekton distribution, a water-mass mixing analysis was carried out as described in Castro et al.^31^ and Machín and Pelegrí^32^. The method assumes that at each depth, the observed temperature (T) and salinity (S) is a linear mixing between three different water types:

1 $${x}_{1}{T}_{1}+{x}_{2}{T}_{2}+{x}_{3}{T}_{3}=T,$$

2 $${x}_{1}{S}_{1}+{x}_{2}{S}_{2}+{x}_{3}{S}_{3}=S,$$

3 $${x}_{1}+{x}_{2}+{x}_{3}=1,$$

where Ti and Si, i = 1:3, are the characterized temperature and salinity values of each water type, and x_i_ are the fractions of each water type contributions. Descriptions of the hydrographic conditions and water masses in the northeast Atlantic can be found in several works, e.g., García-Seoane et al.^21^, Bashmachnikov et al.^33^ and Valdés and Déniz-Gonzaáez^34^. In the study area, we identified five principal water types (Fig. 2a); upper and lower North Atlantic Central Water (NACW), Mediterranean Overflow Water (MOW), Antarctic Intermediate Water (AAIW), and the high-salinity surface and subducted Subtropical Underwater (STUW). Since a maximum of three water types can be used to solve the linear Eqs. (1–3), the area was grouped with three water mass types in each group^32^. For example, the more surface or near-surface upper NACW and STUW will likely not be in contact with the lower layers, AAIW and MOW (see also Fig. 2a). Mesoscale eddies can, however, drive vertical fluxes of water between the surface and the mesopelagic layer (e.g., Della Penna and Gaube^35^), but only one eddy of Mediterranean water at 700–1200 m depth was observed from the CTD data (see the “Results” section). The groups are separated by potential density (σ_θ_): AAIW, MOW, and lower NACW in the lower layer (σ_θ_ > 27.25 kg m^−3^); AAIW, lower and upper NACW in the middle layer (27 < σ_θ_ < 27.25 kg m^−3^); and STUW, lower and upper NACW in the upper layer (σ_θ_ < 27 kg m^−3^). Each temperature/salinity-measurement point was classified with the water type that had the highest contribution given by the water-mass analysis. The reference temperature and salinity values are given in Supplementary Table S1.

### Acoustic measurements

Acoustic data were collected using a Simrad EK80 multifrequency acoustic system comprising six split-beam transducers (operating at 18, 38, 70, 120, 200 and 333 kHz) installed on the vessel’s drop-keel—the transducers were then 11.6 m below the sea surface. Data were collected down to 1200 m depth at 18 kHz and 38 kHz. Due to acoustic absorption, data from higher frequencies do not cover the entire mesopelagic depths and these data are not included here. The drop-keel mounted echosounder was calibrated in January 2019 using standard methods^36^. The resulting configuration and parameters are presented in Table 1. Based on visual scrutiny of acoustic data after the cruise, the study area was divided in five areas based on the different backscatter patterns found in the echograms along the cruise track (Fig. 1).

**Table 1:** Hull-mounted EK80 system settings and calibration parameters on R/*V Kronprins Haakon*.

EK80 system	18 kHz	38 kHz
Transducer		
Model	ES18	ES38B
Equivalent beam angle [dB]	− 17	− 20.7
Calibration		
Gain [dB]	23.0	27
Sa correction [dB]	0.07	0.03
Beams		
Alongship half power opening angle [°]	10.1	7
Offset along. angle [°]	− 0.04	0.03
Athwartship half power opening angle [°]	10.5	7.3
Offset athwart. angle [°]	− 0.03	0.06
Survey settings		
Sound speed [m/s]	1522.1^a^	1522.1^a^
Pulse duration [ms]	1.024	1.024
Transmit power [W]	2000	2000

^a^Before 3 May 23 UTC it was 1472.1.

A trawl-mounted echosounder and a towed platform instrumented with echosounder (MESSOR^37^) were additionally used to sample with higher acoustic frequencies at depth ranges that were not practical from the vessel. A total of 13 daylight stations used a Kongsberg WBAT echosounder mounted on the macroplankton trawl. The WBAT was affixed to the headrope of the trawl and generated either a 120 or 200 kHz forward-facing narrowband pulse with a duration of 128 µs or 256 µs at a ping rate ranging from 1 to 27 s (Table 2). The first four stations were sampled using a 120 kHz transducer but due to technical problems, a 200 kHz transducer was used for the remaining stations. To look at the vertical distribution of mesopelagic organisms in the five areas described above, one trawl from each area was selected. The acoustic target strength (TS) measurement of the micronekton was done with a single-echo detection (SED) method (minimum TS: − 80 dB, min/max echo length (relative to pulse length): 0.6/1.4, maximum phase deviation: 10 phase steps, maximum gain compensation: 3 dB). Echoes from micro and macroplanktonic organisms are also able to be detected with these settings. Detected echoes were converted to densities of organisms following Underwood et al.^38^), and averaged in 40-m depth bins. Density peaks observed in those vertical profiles were assumed to indicate a scattering layer.

**Table 2:** Overview of macroplankton trawl deployments on where a WBAT was attached.

Trawl deployment details						WBAT		
Station	Date	Start time (UTC)	Latitude (N)	Longitude (W)	Max depth (m)	Transducer	Range (m)	Settings
4601	2019-05-03	14:52:07	17.969	− 23.956	1650	ES120-7CD # 105	150	256 µs pulse duration, 400W
4602	2019-05-04	09:30:27	19.513	− 23.174	1200	ES120-7CD # 105	150	256 µs pulse duration, 400W
4603	2019-05-06	09:54:41	24.975	− 20.311	1200	ES120-7CD # 105	150	256 µs pulse duration, 400W
4605	2019-05-08	09:22:51	29.140	− 17.965	1200	ES120-7CD # 105	150	256 µs pulse duration, 400W
4607	2019-05-10	07:55:25	30.612	− 13.590	1200	ES200-7CD #129	100	256 µs pulse duration, 150W
4608	2019-05-11	14:37:09	31.634	− 10.510	1200	ES200-7CD #129	100	256 µs pulse duration, 150W
4609	2019-05-12	08:18:09	32.700	− 11.936	1200	ES200-7CD #129	100	256 µs pulse duration, 150W
4610	2019-05-13	08:20:03	33.695	− 13.232	1200	ES200-7CD #129	100	128 µs pulse duration, 150W
4612	2019-05-15	08:22:33	35.149	− 15.170	1200	ES200-7CD #129	50	128 µs pulse duration, 150W
4613	2019-05-16	09:49:34	36.114	− 16.494	1200	ES200-7CD #129	30	128 µs pulse duration, 150W
4615	2019-05-19	09:26:13	42.982	− 12.318	1200	ES200-7CD #129	30	128 µs pulse duration, 150W
4617	2019-05-21	08:00:52	45.954	− 9.588	1200	ES200-7CD #129	30	128 µs pulse duration, 150W
4618	2019-05-22	07:13:09	47.255	− 8.034	1200	ES200-7CD #129	30	128 µs pulse duration, 150W

To check whether density estimates of the mesopelagic organisms were similar at 120 kHz and at 200 kHz, we compared density profiles estimated simultaneously at the two frequencies, using daytime data from the towed body, MESSOR (n = 3; stations 119, 120 and 128). It was equipped with a four channel echosounder (Simrad EK80 WBT Tubes operating at 38, 70, 120, and 200 kHz). The transducers were mounted on the bottom plate of MESSOR, facing downwards. Only 120 and 200 kHz data are used here. Both transducers were operated in broadband mode (frequency modulated pulses of 93–155 kHz and 160–260 kHz). Sampling range was reduced to 60 m as the reduced power also reduced the signal-to-noise ratio (SNR), hence limiting the working range. Ping intervals ranged from ~ 250 to 350 ms. Only organisms within 4–20 m away from MESSOR and 4–15 m and 3–15 m from the trawl headline (120 and 200 kHz, respectively) were counted. Paired densities estimated from the 120 and 200 kHz transducers on the towed body suggested only very minor differences (200 kHz 3% higher, R^2^ = 0.93) and the values from the two frequencies were used interchangeably (see the MESSOR vertical profiles at these 3 stations in Supplementary Fig. S1).

### Acoustic data processing

All the acoustic data collected during this cruise were analyzed using the Large Scale Survey System (LSSS) postprocessing software^39^. The first step of post-processing of hull-mounted and WBAT data was to apply noise removal filters (details in Supplementary Table S2) to remove backscatter from unwanted sources (e.g., from other acoustic instruments, background noise and on rare occasions false bottom echoes) using the KORONA pre-processor (a component of LSSS). However, applying the filters did not remove all unwanted noise and the data were then visually inspected to remove remaining noise. Only acoustic daylight data were used in the present study. To minimize the impact of crepuscular periods during which micronekton and macrozooplankton ascends and descends (e.g., Irigoien et al.^7^ and Klevjer et al.^17^), only data collected later than 1 h after sunrise and earlier than 1 h prior to sunset were included in the analysis.

Nautical Area Scattering Coefficient (NASC, m^2^ nmi^−2^) values^40^ from 15 to 1200 m depth were calculated for every integration cell (of size 5 m in depth and 600 s temporally) in the whole survey transect for the hull-mounted 18 and 38 kHz with an *S*_*v*_ threshold of − 85 dB re 1 m^−1^. Due to the drop keel depth and the near-field of the echosounder transducers, the upper 15 m of the water column was not available or included in the analysis. After echo-integration, backscatter values were corrected for errors caused by static absorption/sound speed profiles utilized in LSSS, using the same methods as Haris et al.^41^. DSL widths were plotted in the echograms and were calculated from the full-width-half-max value (i.e., the width of the signal at half the maximum amplitude).

### Abundance models

Abundance of micronekton was estimated using two different approaches: (1) using NASC collected with hull mounted echosounders at 18 and 38 kHz as proxy, (2) by echo counting using high frequency (120 and 200 kHz) data obtained from the trawl-mounted echosounder (WBAT). We assumed that the micronekton dwelling in the surface layers during daylight and those in the deeper waters differ and thus, their response to environmental variables could also be different. We therefore split the estimated abundances (both NASC and echo counts) into two data sets: surface (0–200 m depth), and deep layers (from 200 to 1200 m depth). The covariate water masses were considered as a factor and in the WBAT datasets (both surface and deep layers), the STUW level was excluded from the analyses because of too few observations. For the NASC dataset, only data from times of 7:00–18:00 were included in the models in order to explore the interaction term between water masses and time. In addition, fluorescence was omitted from the models because the micronekton in general does not feed on chlorophyll directly, and instantaneous levels of chlorophyll are expected to be a poor predictor of time-integrated levels (e.g., annual or seasonal primary production) (e.g., Geider^42^, Westberry et al.^43^).

Data exploration was conducted following the protocol described in Zuur et al.^44^ for both approaches. Two outliers were detected in the NASC values from deep layers at 18 kHz and discarded from the analyses. High collinearity was detected between the water masses and both environmental variables (temperature, salinity and oxygen) and depth. Thus, the final models included environmental variables and depth as proxies for the water masses because abundance of micronekton modelled as function of the environmental variables and time, showed lower AIC values and much higher deviance explained than the ones using as explanatory variables water masses. Before the analyses, collinear covariates were removed considering the correlation between variables and the variation inflation factor (VIF) using the cutoff value of 3^44^. We used AIC for model selection and an assessment of residuals for model validation.

Generalized Additive Models (GAMs)^45^ were applied to assess possible non-linear effects of the environmental factors (temperature, salinity and oxygen) and depth in the abundance of the micronekton. For NASC models in deep layers high values of concurvity was detected between temperature and salinity and for the echo count model from surface layers between temperature and oxygen. Concurvity leads to significance tests with inflated type I error, which might result in concluding that the results are statistically significant when no effect exists^46^. Thus, we excluded salinity and oxygen from these models. To deal with zeros and due to the data set showed large dispersion, a tweedie distribution was applied^47^. We used AIC for model selection and an assessment of residuals for model validation. For estimating the model coefficients and the smoothing parameters of the GAMs, restricted maximum likelihood (REML) was used. Thus, the final models were:

$$\text{Abundance(NASC)}=Time \; of \; the \;day+s\left(Temperature\right)+\text{s(Depth)+s(Oxygen)}+\varepsilon$$

for micronekton from surface.

$$\text{Abundance(NASC)}=Time \; of \; the \;day+s\left(Temperature\right)\text{+s(Oxygen)}+\varepsilon$$

for the micronekton from deep layers.

$$\text{Abundance(echo counts)}=Time \; of \; the \;day+s\left(Temperature\right)+\text{s(Depth)}+\varepsilon$$

for micronekton from surface.

$$\text{Abundance(echo counts)}=Time \; of \; the \; day+s\left(Temperature\right)+\text{s(Salinity)+s(Oxygen)}+\varepsilon$$

for the micronekton from deep layers.

All the variables except time, which was treated as a factor, were smoothed. The smoothers used in the models were thin plate regression splines. In preliminary analyses, the geographical position (UTM coordinates) was included in the models using a two-dimensional tensor smoother for the NASC models and as a random effect for echo counting models. We decided to not include these covariates in the final models because they showed strong concurvity (> 0.8) with some environmental variables (temperature for surface layers in the NASC models, oxygen for all deep layer models and oxygen and temperature for echo count models from surface layers). In addition, the covariate geographical position was not sufficient to model all the spatial correlation and these models had higher AIC values than the model without geographical position. Thus, in the models there remained some spatial autocorrelation at small scale in the Pearson residuals, except for the echo count model from deep layers (Supplementary Fig. S2). We accepted this spatial correlation in the residuals in our final model because the tweedie distribution was not implemented in the R package used for dealing with it (R-INLA version 20.07.12).

To analyze and visualize the data MATLAB (MathWorks Inc.) and R software (R Development Core Team 2022) were used. The packages “fields”^76^, ggplots2^77^ and “lattice”^78^ were used for constructing the graphs, the package “reshape2”^79^ and “plyr”^80^ for data manipulation, the package “maptools”^81^ for estimating sunrise and sunset times and the package “mgcv”^82^ for GAM fitting and “sp”^83,84^ for spatial coordinates manipulation.

### Ethical approval

No approval of research ethics committees was required because no animals has been handled to accomplish the main goals of this study. The trawling (in which the WBAT was attached) and the subsequent biological sampling, has been conducted within the framework of a scientific survey.

## Results

### Environmental variables

**Figure 3:**
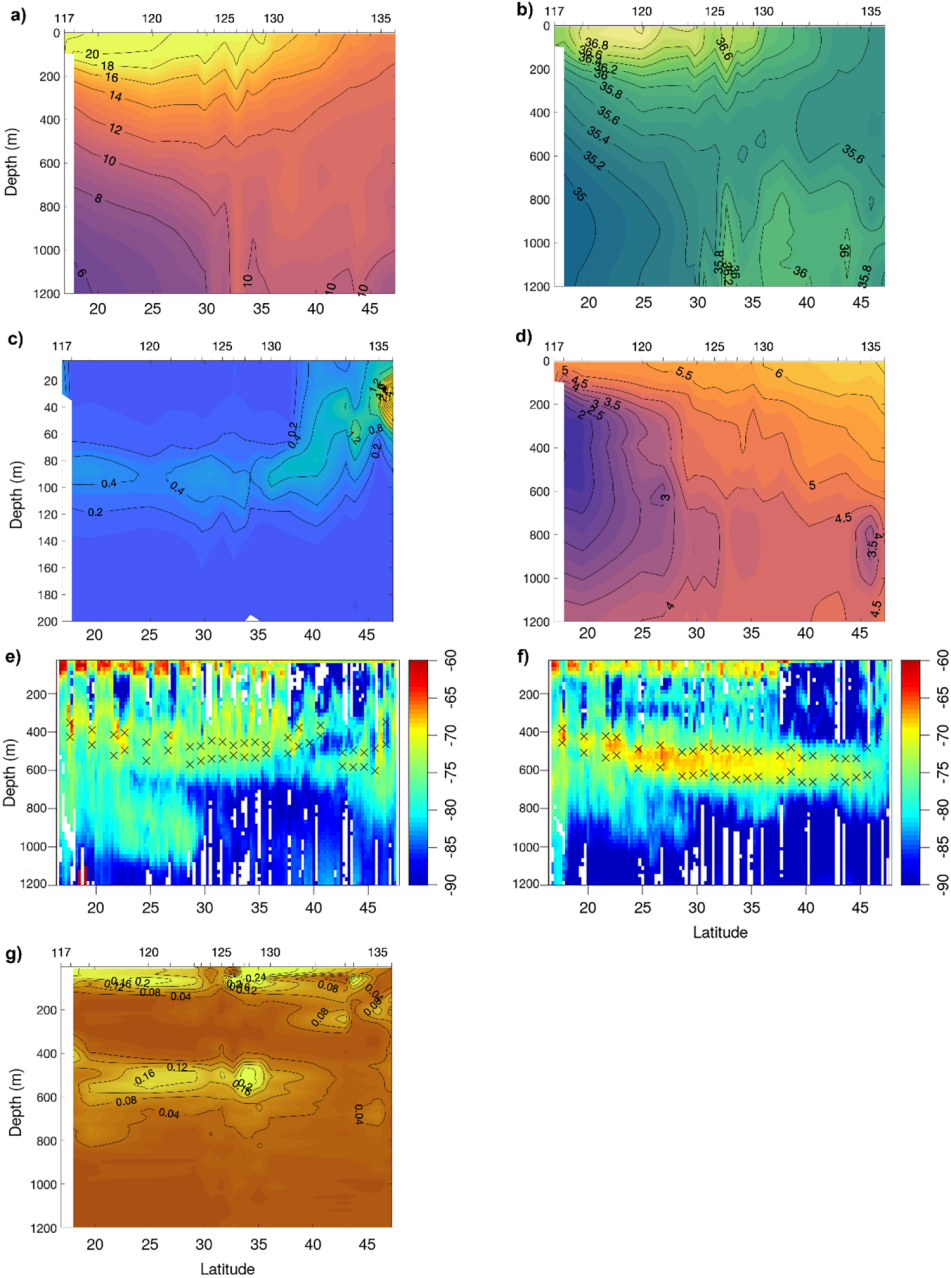
Latitudinal and vertical variation during the cruise transect in (**a**) temperature (°C), (**b**) salinity, (**c**) fluorescence (mg m^−3^), (**d**) oxygen (ml l^−1^), volume backscattering strength (*S*_*v*_) (dB re 1 m^−1^) (**e**) at 18 kHz and (**f**) at 38 kHz, (**g**) micronektonic densities (number of echoes m^−3^) estimated using trawl mounted WBAT (120 and 200 kHz). In these plots, the values between stations, with the exception of (**e**) and (**f**), were linearly interpolated between the values at each pair of stations. Note that in (**e**) and (**f**) while all daytime data (sun above horizon) is plotted in the echograms, only data for periods where the sun was more than 50 degrees above horizon were included in the calculation of DSL widths (full-width-half-max relative peak DSL *S*_*v*_ levels, marked by X) to reduce the impact of vertical migration on the estimates. For the rest of the graphs, the position of the CTD station numbers is indicated in the upper part. This figure was created using MATLAB, version R2018a from MathWorks (https://www.mathworks.com) and R version 4.1.3 (2022-03-10) (https://www.r-project.org/).

The lower surface temperature (~ 18 °C) along the northwestern coast of Africa due to coastal upwelling of colder water masses was clearly observed-station number 125 was located within this upwelling (Fig. 1). Down to 200 m depth, relatively high temperature waters were observed south of 30° N (Fig. 3a). In addition, from 30° N and northwards there was a weaker vertical stratification in the upper 500 m, compared to 30° N and southwards, where the temperature decreased strongly with depth, from 12 °C at 400 m depth to 6 °C at 1200 m depth. From ~ 30° N and northwards, a homogeneous layer of oceanic waters (in terms of temperature) was observed at 300–1100 m depth with temperatures around 11 °C.

Relatively high salinity waters were also observed from down to 200 m depth in the southern part (from 30° N and southwards), with the maximum salinity above 37 psu at 20–25° N (Fig. 3b). North of 30° N, the salinity showed little variation with depth in the upper 700 m in contrast to the salinity variation observed south of 30° N.

There, the salinity was below 35 psu at 800–1000 m depth and was the absolute salinity minimum in the area. North of 30° N and below 700 m depth, the salinity was relatively high and above 35.8 psu. At station number 126, a local maximum in salinity (36.2 psu) was observed at 700–1200 m depth.

The observed chlorophyll concentrations at upwelling stations did however not show enhanced production in the upwelling region (Fig. 3c). In fact, fluorescence values showed an increase in the first 100 m from 37° N (with values > 1 mg m^−3^) reaching the maximum values of observed (above 4 mg m^−3^ at the surface around 45° N (Fig. 3c)).

The main characteristic of the oxygen concentrations was the minimum at 200–600 m depth in the south, at station numbers 118–119, with values below 2 ml l^−1^ (Fig. 3d). The observed area of reduced oxygen values is part of the larger oxygen minimum zone in the eastern North Atlantic (e.g., Stramma and England^48^).

### Water masses

The characteristic temperature and salinity values for each water type made it possible to determine the different water masses in the section (Fig. 2b). The lower NACW was found in all stations and occupied most of the region, especially at 200–700 m depth. South of about 35° N, STUW was the dominant water type in the upper 200 m, with intrusion of upper NACW mainly at the southernmost stations 117–119. At intermediate levels (below 700 m depth), the AAIW occupied the southern part, to about 30° N while MOW was the dominant deep water type north of 30° N, with the presence of a “meddy” between 33 and 35° N.

At both frequencies, high backscatter values were associated with STUW and u-NACW (Table 3), which are the water masses located south of 37° N close to surface waters (0–200 m depth). Considering the average backscatter of each water mass, at 18 kHz, the u-NACW showed the highest average backscatter (28.02 m^2^ nmi^−2^) while at 38 kHz it was the STUW (20.69 m^2^ nmi^−2^). However, the maximum values of backscatter for both frequencies were found in the STUW (5027.36 and 5124 m^2^ nmi^−2^, respectively). The lowest backscatter was found in deep waters (~ 800–1200 m depth). The low values of average backscatter at each waters mass were found in MOW (0.43 m^2^ nmi^−2^ at 18 kHz and 0.16 m^2^ nmi^−2^ at 38 kHz) followed by the AAIW (1.99 m^2^ nmi^−2^ at 18 kHz and 0.73 m^2^ nmi^−2^ at 38 kHz).

**Table 3:** Mean NASC values in the water column (from 15 to 1200 m) according to the oceanographic zones for different frequencies.

Oceanic zones	18 kHz			38 kHz		
	Mean NASC m^2^ nmi^−2^ (standard deviation)	Max NASC	n	Mean NASC m^2^ nmi^−2^ (standard deviation)	Max NASC	n
AAIW	2.0 (2.1)	14.7	21,597	0.7 (1.3)	14.0	22,559
l-NACW	6.1 (19.0)	4193.4	146,766	7.4 (14.8)	1191.3	150,605
STUW	9.9 (114.6)	5027.4	21,501	20.7 (175.5)	5124.8	22,905
MOW	0.4 (0.7)	115.2	51,162	0.2 (0.3)	11.9	51,479
u-NACW	28.0 (35.6)	582.0	3703	9.9 (21.6)	548.7	3717

AAIW = Antarctic Intermediate Water, l-NACW = lower North Atlantic Central Water, STUW = Subtropical underwater, MOW = Mediterranean Overflow Water and u-NACW = upper North Atlantic Central Water). N = sampling size of the NASC values, i.e., the number of integration cells (NASC in each 5 m depth and 600 s temporally) corresponding to each water mass.

### Horizontal and vertical acoustic distribution of micronekton across latitudes

The vertical profiles of *S*_*v*_ from both frequencies (18 and 38 kHz), as well as the directly estimated echo densities using higher frequencies (120 and 200 kHz), illustrate the spatial variation in horizontal and vertical distribution of the micronekton (Fig. 3e,f,g). Several mesopelagic DSLs were seen at both 18 kHz and 38 kHz, with relative vertical distributions showing similar horizontal patterns at both frequencies. However, at 18 kHz (Fig. 3e), the upper DSL (~ 400–600 m depth) was weaker and with boundaries located around 50 m shallower than the one at 38 kHz. Furthermore, the lower DSL (~ 600–1000 m) had higher backscatter levels at 38 kHz than at 18 kHz. In general, the estimated densities by echo counting (using trawl-mounted echosounder data from 120 and 200 kHz) were highest from 0 to 100 m depth and at around 500 m depth (Fig. 3g). However, the overall micronektonic densities estimated by echo counting decreased from 37° N northwards, in synchrony with the backscatter in the scattering layers. Considering these vertical changes in *S*_*v*_ distribution along the survey track we defined five areas:

- Area 1 (from 17 to 18° N): was characterized by two intense deep scattering layers at mesopelagic depths at 38 kHz: one around 400 m (which was the most intense) and the other around 650 m (Fig. 3f). A peak in echo densities was also detected by the WBAT at mesopelagic depths (0.15 m^−3^ at 400 m depth) (Fig. 3g).
- Area 2 (from 18 to 30° N): is a transition area. The trend for the deep scattering layers (both at 18 and 38 kHz) is increasing depth from the beginning of the cruise until 30° N (Fig. 3e,f). At 38 kHz, the upper bound of the DSL deepened from around 400 m in the south to around 500 m at 30° N. For all areas north of 30° N the upper bound of the DSL was located around 500–550 m (Areas 3,4 and 5). Echo densities (120 or 200 kHz) in the depth range of the deeper DSL were low (Fig. 3g).
- In Area 3 (from 30 to 37° N), the WBAT estimated densities in the mesopelagic layer peaked (0.2 echoes m^−3^) (Fig. 3g). The lower DSL was absent from this area (i.e., from 30° N and further north) on both frequencies (18 and 38 kHz). A shallower scattering layer (located from 200 to 300 m depth) was present along much of the track at 38 kHz, but had increased levels of backscatter in this area.
- In Area 4 (from ~ 37° N to 45° N), the vertical distribution of the micronekton was structured differently: the backscatter energy in the ~ 0–100 m depths was lower than in the rest of the cruise track. The mesopelagic backscatter energy and echo densities were, in general, also low. The shallower scattering layer at 38 kHz (200 m depth) was absent in this region and the maximum densities recorded in the area were at 250 m depth.
- In Area 5 (from 45 to 48° N), the backscatter in surface waters (0–100 m) and the shallow scattering layer (200 m) were present again (Fig. 3e,f). The echo densities below 300 m are also low, although they are slightly higher than in Area 4. A peak of increased densities of organisms was observed between 150 and 200 m depth.

**Figure 4:**
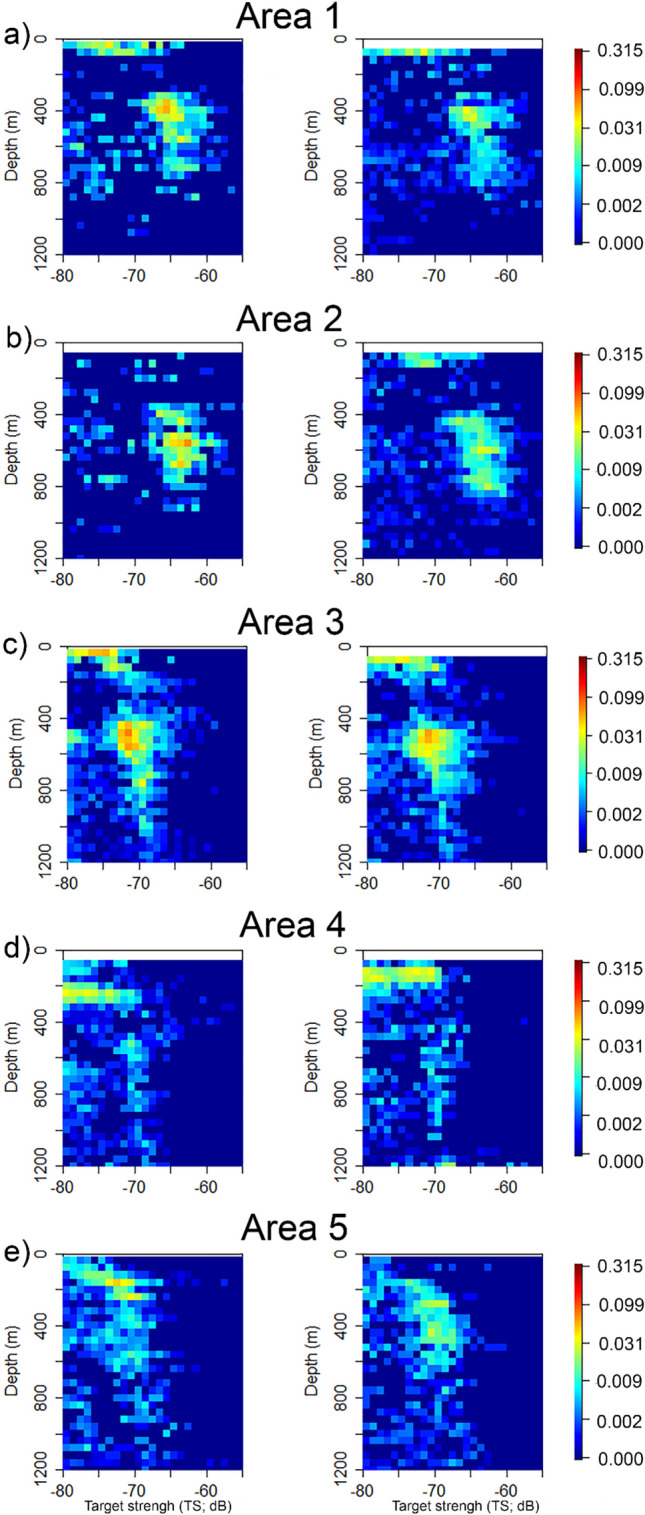
Example of the vertical profiles (down cast on the left and up cast on the right) of TS and densities of echoes detected by the WBAT mounted in the trawl per area. The colour scales indicates the density (number of organisms m^−3^). This figure was created using R version 4.1.3 (2022-03-10) (https://www.r-project.org/).

The vertical distribution of measured target strength (TS) (at 120 and 200 kHz) also varied with latitude (Fig. 4). From 17 to 37° N (i.e., Areas 1, 2, and 3), higher densities of echoes in the surface layers were mainly associated with weaker TS values (Fig. 4a–c). In contrast, the TS composition inside the depth ranges occupied by DSLs was mainly associated with stronger TS. In Area 4, echo densities inside the layers were lower and were accompanied by vertical changes in the TS distributions (Fig. 4d). The peak densities of echoes in that area were detected around 100–300 m and corresponded to targets with TS values between − 80 and − 70 dB re 1 m^2^. In Area 5, the echo densities were also lower than in Areas 1, 2 and 3 and peak densities of mesopelagic organisms were distributed between 100 and 600 m depth (Fig. 4e). High densities of echoes were absent in the surface layers, with the few organisms present having higher TS values (> − 75 dB). In these two areas (4 and 5), the vertical TS profiles were different between downcast and upcast, e.g., the depth of peak densities of echoes would shift (Fig. 4).

### Abundance of micronekton and macroscale oceanography.

The location of the DSLs coincided with isotherms (Fig. 3). The upper DSL at both frequencies was situated in waters masses at approximately 11 °C along the whole cruise track, while the lower scattering layer tended to distribute between 7 and 9 °C. From 44 to 45 °C, the upper DSL increased in thickness, from being at ~ 400–600 m to being situated ~ 200–800 m depth, coinciding with waters of homogeneous salinity around 35.6 psu (Fig. 3b).

**Figure 5:**
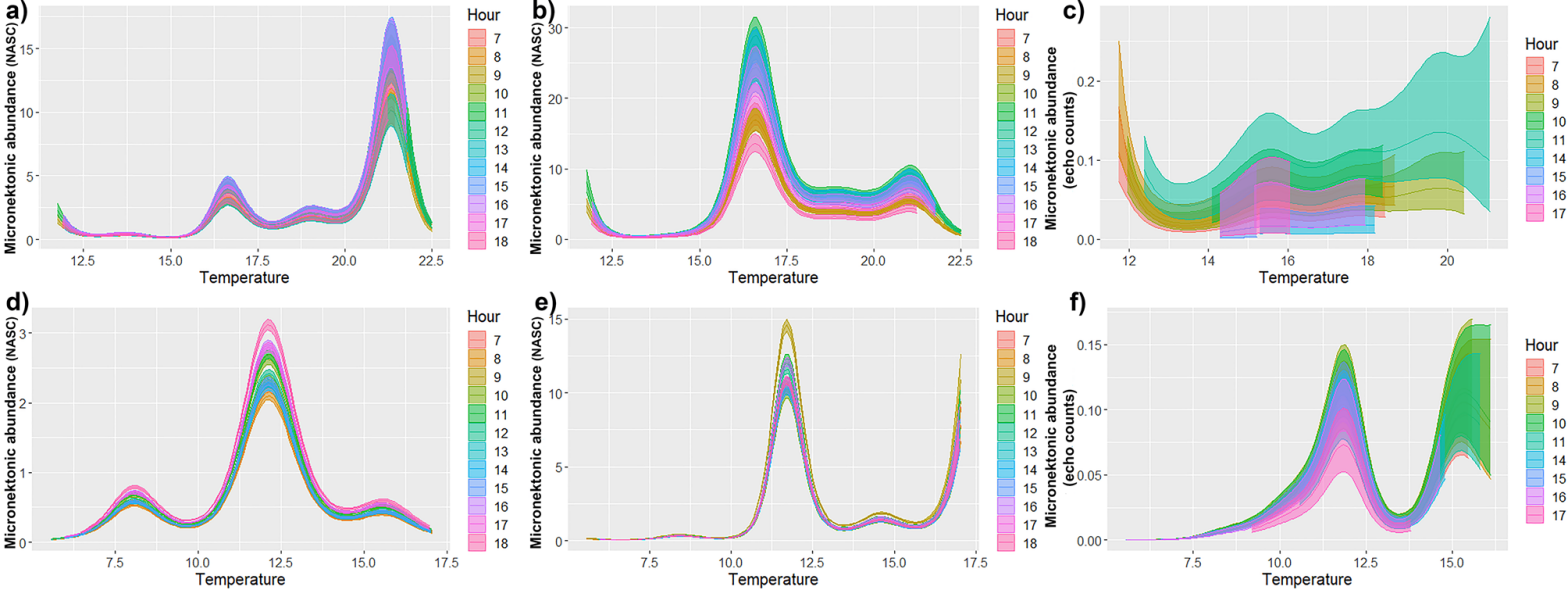
Fit of the of the temperature per hour for (**a**) average depth and oxygen in micronekton from surface at 18 kHz, (**b**) for average depth and oxygen in micronekton from surface at 38 kHz (**c**) for average depth in micronekton from surface at high frequency, (**d**) for average oxygen in micronekton from deep layers at 18 kHz, (**e**) for average oxygen in micronekton from deep layers at 38 kHz and (**f**) for average salinity and oxygen in micronekton from deep layers at high frequency. The upper and lower lines for each time of the day represent the 95% confidence intervals around the fitted values (middle line). This figure was created using R version 4.1.3 (2022-03-10) (https://www.r-project.org/).

We analyzed the NASCs (as a proxy for micronekton abundance) and echo densities (measured with high frequencies) in relation to the hydrography and time of day (i.e., hour) for micronekton from surface and from deeper layers as separated datasets. The covariates (the environmental factors, depth and time of the day) had a significant influence on the backscatter (NASCs) at both frequencies (18 and 38 kHz) and in the densities based on echo counts at 120/200 kHz for both data sets (surface and deep layers) (Supplementary Table 3). The deviance explained by the GAM for near surface micronekton (0–200 m) was 47.8%, 51.7% and 31.9% (at 18 kHz, 38 kHz and 120/200 kHz, respectively) and for micronekton from deeper layers was 45.1%, 67.0% and 58.2% (at 18 kHz, 38 kHz and 120/200 kHz, respectively). The tweedie parameter was estimated to be 1.87, 1.88 and 1.51 for surface’s micronekton and 1.86, 1.77 and 1.32 for deep layers’ micronekton (at 18 kHz, 38 kHz and 120/200 kHz respectively). The smooth functions fitted for both models suggested non-linear responses to the environmental variables and depth. In the surface waters (0–200 m), the NASC values predicted by our model mainly peaked around 21 °C at 18 kHz (Fig. 5a) and around 17 °C at 38 kHz (Fig. 5b). In contrast, for surface waters the highest predicted densities of echoes were associated with lower temperatures (~ 12 °C), and these densities decreased for temperatures from 12 to 14 °C, and afterwards tended to increase with increasing temperatures (Fig. 5c). In the deep layers, the 18 kHz modelled effect of the temperature on the backscatter was dome shape from ~ 10 to 14 °C and it showed a small peak around 8 °C (Fig. 5d), while for the 38 kHz model the predicted NASC values also peaked around 11 °C (but this peak was narrower than in 18 kHz) and they increased again at higher temperatures (~ 17.5 °C) (Fig. 5e). For the echo counts, this peak on predicted densities around 11 °C was also present, and densities increased with temperature from 14–15 °C, reaching a plateau for the higher temperatures (Fig. 5f).

## Discussion

In this work, we modeled the influence of environmental variables on micronektonic abundances. Continuous variables (such as temperature and salinity) are more flexible than categorical variables (e.g., water masses), and as a result, the models with environmental variables explained higher variability than the models using water mases as explanatory variables. The influence observed in the data are statistical relationships and do not necessarily imply that it reflects cause-effect relationships. In fact, several of these environmental variables are proxies for water masses. For instance, both temperature and salinity are highly correlated with the water masses. The implications are that instead of looking for an optimum temperature, micronekton could be associated with waters masses for historical reasons and move with them. Hence, the water mass is the biotope for plankton communities, not the temperature/salinity range^49^. Cook et al.^50^ suggested that the biological history of a body of water mass is more important for species assemblage than the physical and chemical features of the water masses. The occurrence or exclusion of non-migratory mesopelagic fishes might be associated with particular water masses. On the other hand, migrant mesopelagic fish occur in a wide depth range and travel through different water masses during their vertical migration, thus, it is more difficult to establish this correlation, although some migrant species have been correlated to the Eastern NACW (ENACW^51^).

At 18 kHz, some backscatter from the lower DSL is associated with the AAIW, whereas this layer is absent in MOW. Olivar et al.^51^ observed that *Cyclothone pallida* occurred at only stations at depths with AAIW. AAIW is an oxygen and silica rich water mass, but the oxygen concentration decreases as it flows northward from the Antarctic Circumpolar Current and along the African northwest coast to 30–32° N^32,52^, and it has a salinity minimum between 500 and 1200 m depth^48^. MOW flows out through the Strait of Gibraltar, occupies the 700–1500 m depth, and is characterized by high salinities and temperatures^53^ but with low oxygen and nutrient contents^33^. The DSL between 400 and 700 m depth over the whole transect (Fig. 3e,f) is located within the NACW. Olivar et al.^51^ detected *Cyclothone braueri* only at the same depth range at stations with 100% ENACW. NACW is the principal water mass in the upper layer of the North Atlantic (e.g., Bashmachnikov et al.^33^), and in the transect it reached down to about 800 m depth.

A strong coupling between backscatter and water masses has been reported in the Norwegian and Greenland Seas, with higher backscatter values associated with the relatively warm Atlantic Water, whereas the colder Arctic Intermediate Water was almost devoid of backscatter^54^. Strong correlation between the horizontal and vertical distribution and the water masses has also been found in the South-Western Indian Ocean, and the total backscatter peaked in the subtropical zone with low abundance in the colder Polar Frontal Zone^55^.

### Factors influencing large scale distribution

For most of the cruise (except from 37° N to 45° N), the highest backscatter at 18 kHz was found in near-surface waters, whereas at 38 kHz, the upper DSL and near-surface waters have similar backscatter levels. In this study, the location of the deep scattering layers coincided with isotherms, suggesting a tight linkage between daytime DSL depth and temperature. The micronekton that compose the upper (and stronger) DSL at both frequencies coincided with waters of 11 °C regardless of distance from the equator, while those from the lower DSL live in temperatures around 7–9 °C. This observation is reflected in our models from deep layers (both at high and low frequencies), showing micronektonic abundances peaking around 11 °C. In addition, a backscatter peak was also found at 8 °C in the 18 kHz deep layers model, coinciding with the lower DSL. Temperature plays an important role in the distribution patterns of the marine species: temperature directly affects metabolic rates and thus have a direct impact on individual growth and reproduction^56^. During this cruise, a significant relationship between the fish assemblages and the temperature in the water column was also reported^21^. Fish communities showed a pattern of lower diversity in cold and more productive waters (from 42 to 48° N) and an increase in diversity in warmer conditions (from 27 to 37° N)^21^. In terms of densities, *Cyclothone* species were the main species for both fish communities collected in this cruise, while *Benthosema glaciale*, *Maurolicus muelleri*, and *Xenodermichthys copei* were only characteristic of colder waters (i.e., from 42 to 48° N)^21^.

In the northernmost part of the study area, DSLs were located inside layers of homogeneous temperature (waters of 10–12 °C between ~ 300 and ~ 1000 m depth), pointing towards factors other than temperature as being important in shaping both spatial and vertical micronekton distributions. This is supported by our models, which also showed that salinity, oxygen and depth were statistically significant variables. The lowest backscatter was found in the MOW, and the high salinities from this water mass also corresponded with the absence of the lower DSL. These facts suggest that salinities might indicate low micronekton abundances as a result of advected MOW instead of the salinity affecting the micronekton distribution.

In the Cape Verde region, we observed strong backscatter layers in the oxygen minimum zone. A previous study was not able to find any significant relations between fish density and biomass and oxygen levels averaged over parts of the water column^21^. Some non-migratory fishes (e.g., *Cyclotone* spp. and *Sternoptyx diaphana*) may live in low oxygen concentrations both day and night^51^, and high concentration of *Cyclothone* species have been reported in OMZs from different oceans^51,57^. Oxygen depleted zones influence the distribution of most midwater species (both vertically and horizontally), and some OMZ’s may support highly specialized organisms^58^. For example, fauna living in OMZ typically show adaptations, such as large gill surface area or thin membranes, giving short blood-to-water distance^59^. Thus, the low oxygen concentrations reported in our work (< 2 ml l^−1^) could be acting as a refuge from predation pressure^60^, similar to what has been found in other areas. The observed oxygen levels were below the hypoxic threshold of some predators, such as skipjack tuna and istiophorid billfishes (< 3.5 ml^−1^)^61^. During this cruise, the micronekton densities measured in the OMZ were quite high and there was a significant relationship between organism density and oxygen levels. Low oxygen concentrations will alter the species composition but may also affect the acoustic properties of the organisms subjected to the low concentrations steadily decreasing the mass of gas in the fish swimbladder, even on relatively short timescales^62^, thus, the relationship between abundances/biomass and backscatter may differ between low and high oxygen areas.

We restricted our analysis to daytime data only to avoid confounding patterns of changes in micronekton distribution due to diel vertical migrations. However, time of day was significant, suggesting that some variability is explained by the hour, which agrees with the light comfort zone hypothesis. This hypothesis proposes that micronekton occupies a light comfort zone that avoids both strong and low light intensities^13,14^. In addition, light intensities to which micronektonic organisms are exposed depend on the hour of the day. The variability in the oceanic DSL daytime depth is controlled by variation in light penetration, but oxygen levels are also important^14,16^. Micronekton, in particular krill and myctophids, take refuge not only in the dark waters located at mesopelagic depths, but also in areas with low oxygen levels^63^. Recent large-scale studies reported that DSL daytime depth was linked to oxygen levels^15^.

A previous study^20^ used observed acoustic patterns to assign “biogeographical” regions, but acoustic patterns also reflect behaviour, so it is unclear what the resulting “biogeographical” regions represent. In our case, changes in acoustical patterns did not coincide with changes in fish communities. Our study covered three of the 33 ecoregions defined by Sutton et al.^19^: Mauritania/Cape Verde, Central North Atlantic and North Atlantic drift, belonging to three different types of biomes. We were unable to identify these three regions from the observed patterns in vertical distribution of backscatter. The Mauritania/Cape Verde biome is an upwelling region, where the oceanic circulation interacts with continental topography and coastal winds leading to upwelling. North of Mauritania/Cape Verde, the source of the upwelling water is the low nutrient NACW^19^. The Central North Atlantic ecoregion is a trade wind biome with low productivity, weak seasonality, and a persistent deep maximum in chlorophyll^19^. The North Atlantic drift ecoregion is, in contrast, a westerly wind biome, with large seasonal changes in mixed layer depth due to high westerly wind stress in winter. It is characterized by large spring algal blooms^19^. The Mauritania/Cape Verde regions include relict and endemic species characteristic of “cool” waters whereas the North Atlantic drift constitutes an admixture of boreal and subtropical species^19^. Different micronektonic communities inhabit these three ecoregions, and trawl catches from our cruise found that fish community structure mirrored the ecoregions^21^. Based solely on observed patterns in the vertical distribution of backscatter, we identified five distinct regions described here, which did not conform well to the ecoregion boundaries. Area 1 (characterized by two intense DSLs) is included in the Mauritania/Cape Verde whereas Area 2 (with DSLs increasing in depth with increasing latitudes) straddles 2 ecoregions (Mauritania/Cape Verde and Central North Atlantic). Area 3 (characterized by only one DSL) and the first part of Area 4 (characterized by the weaker intensity in the scattering layers without layers in surface) belongs to the Central North Atlantic ecoregion while the last part of Area 4 and Area 5 (characterized by still weak intensity in the layers but with a scattering layer in surface waters) belongs to the North Atlantic drift. In Area 4, the catches of salps were high, suggesting that a salp bloom^64^ was occurring. In the area of this salp bloom, DSLs below 300 m were absent. Thus, within a single ecoregion, identified both by previous studies^19^ and verified by catches during our survey^21^, the assemblage of micronekton species may alter their behavior over their distribution range. Differences in vertical distribution of the scattering layers within the same ecoregion might be a reflection of the different migration behaviour of micronektonic organisms belonging to the same species assemblage. For example, different migration amplitude might lead to a different vertical structure of micronektonic organisms, and thus, of the scattering layers. This fact could explain the differences in biogeographic classifications based on taxonomy (e.g., Sutton et al.^19^) and based on acoustical patterns (e.g., Proud et al.^20^), raising the question what we should use to define boundaries of the ecoregions: the species assemblage or the behavior of the species within it?

In the southern part of the study area (from 17 to 37° N), we detected scattering layers in the surface waters, which were mainly absent in the north. These layers were composed of organisms with low TS, most likely crustaceans, gelatinous organisms and/or small fish. Nevertheless, the composition of the DSLs is mainly associated with organisms with higher TS (> ~ − 70 dB), indicating that the composition in those layers was mainly aggregations of gas-bearing organisms, such as small mesopelagic fish. This is supported by the fact that the lower DSL was stronger at 18 kHz than at 38 kHz—small mesopelagic gas-bearing fishes have a resonant backscatter frequency closer to 18 kHz than 38 kHz. On the other hand, in the north of the study area (37–48° N), the echo densities ahead of the trawls were generally low and the target strength distributions (Fig. 4) suggest that the DSLs contained a mixture of strong (mesopelagic fish) and weak scatterers (crustaceans and swimbladderless fish). Trawling and optical data at depth suggested low densities of siphonophores in the area and the strong scatterers are unlikely to be dominated by siphonophores^65,66^. The dominant fish in our catches were members of the gonostomatid genus Cyclothone. *Cyclothone braueri*, *Cyclothone microdon*, *Cyclothone pseudopallida*, and *Cyclothone pallida* collectively made up more than 78% of the organism density in the trawl catches, while the most diverse family was Myctophidae^21^. Other studies in the North Atlantic have indicated that DSLs are dominated by Myctophidae and Stomiiformes^22,67,68^, but many of these studies are based on catches from graded trawls that severely underestimate the densities of *Cyclothone* spp.^51^. Given that catches were completely numerically dominated by *Cyclothone* spp.^21^, and that we lack knowledge of acoustic properties of most of the organisms encountered in the mesopelagic zone during the cruise, we attempted no identification beyond stating that the majority of targets at mesopelagic depths probably originated from fishes, given the TS (Fig. 4) and the general lack of observations of physonect or cystonect siphonophores during the cruise^66,69^.

### Methodological caveats

In the present study, we applied the echo counting method using data at 120 and 200 kHz to provide vertically resolved abundance estimates from daytime trawl hauls. This method was used to obtain high-resolution vertical distribution data on TS as a proxy for organism type (strong vs. weak scatters) and is thus a coarse approach at obtaining information about biological composition in the mesopelagic zone^1,65^. However, echo counting may underestimate the true numerical densities if avoidance is an issue, and increasing sampling volume with range may decrease the probability of detecting echoes at larger distances than this^1^. In contrast to other studies (e.g., Kaartvedt et al.^70^), Underwood et al.^38^ did not observe large avoidance of mesopelagic organisms inside a range of 30 m in front of the macroplankton trawl opening. However, micronekton avoidance might occur beyond 30 m.

Mesopelagic micronekton are commonly mapped at low to medium frequencies (typically 38 kHz) (e.g., Irigoien et al.^7^ and Béhagle et al.^55^), as sound-absorption imposes constraints on signal to noise ratios at mesopelagic depths for higher frequency hull-mounted transducers. The large-scale application of high-frequency data to estimate micronekton densities at depth is a major step forward in mesopelagic mapping, as it largely avoids the resonance issues while also being able to map weak scatterers, which form an important part of the total micronekton biomass^71,72^. The WBAT results complemented the hull-mounted data, which provided an overview of large-scale spatial distribution patterns. While hull-mounted low frequency echosounders permit a rapid and time-efficient mapping of mesopelagic backscatter, the general lack of knowledge of size distributions and acoustic properties of mesopelagic organisms, and largely unknown magnitude of resonance effects makes conversions into biomasses or abundances highly uncertain (e.g., Irigoien et al.^7^, Proud et al.^8^, Godø et al.^73^, Davison et al.^74^). When using low frequency data, stronger scatters, such as swimbladdered fish, will dominate the backscatter, especially when the frequency used is close to the resonant frequency of the swimbladder, and weaker scatters such as crustaceans, gelatinous organisms and fish without swimbladders will be masked (e.g., McClatchie and Coombs^75^ and Davison et al.^74^). Trawl catches can provide size distribution and taxa composition, however, sampling mesopelagic micronekton using trawls is complicated due to net avoidance^70^ and mesh extrusion^51^.

## Conclusion

We measured backscatter with low frequency (18 and 38 kHz) and estimated micronektonic densities with high frequency (120 and 200 kHz) acoustics over a large geographic region. We observed a widespread layered daytime distribution of the micronektonic organisms across the North Atlantic Ocean, from the surface to 1200 m depth, and described the overall latitudinal pattern (from 17 to 48° N) in backscatter and echo densities and its relation to hydrography. Temperature, oxygen and salinity, as well as depth and time of the day, showed a statistically significant influence on the backscatter and echo densities. However, the biotope of the micronekton may be the water masses instead of an optimum temperature/salinity, as has been reported for plankton communities^49^. This study complements the classical estimation of micronekton (using hull-mounted echosounders) with abundance estimations from high frequency acoustic methods (using an echosounder deployed on the trawl), and it increases our knowledge of their spatial variability in the Northeast Atlantic Ocean.

## Supplementary Information

Supplementary Information.

## Data Availability

The data underlying this article will be shared on reasonable request to the corresponding author.

## References

[1] Kloser RJ, Ryan TE, Young JW, Lewis ME (2009). Acoustic observations of micronekton fish on the scale of an ocean basin: Potential and challenges. ICES J. Mar. Sci..

[2] Lehodey P (2015). Optimization of a micronekton model with acoustic data. ICES J. Mar. Sci..

[3] Drazen JC, De Forest LG, Domokos R (2011). Micronekton abundance and biomass in Hawaiian waters as influenced by seamounts, eddies, and the moon. Deep Sea Res. Part I.

[4] Hidaka K, Kawaguchi K, Murakami M, Takahashi M (2001). Downward transport of organic carbon by diel migratory micronekton in the western equatorial Pacific: Its quantitative and qualitative importance. Deep Sea Res. Part I.

[5] Davison PC, Checkley DM, Koslow JA, Barlow J (2013). Carbon export mediated by mesopelagic fishes in the northeast Pacific Ocean. Prog. Oceanogr..

[6] Hidalgo M, Browman HI (2019). Developing the knowledge base needed to sustainably manage mesopelagic resources. ICES J. Mar. Sci..

[7] Irigoien X (2014). Large mesopelagic fishes biomass and trophic efficiency in the open ocean. Nat. Commun..

[8] Proud R, Handegard NO, Kloser RJ, Cox MJ, Brierley AS (2019). From siphonophores to deep scattering layers: Uncertainty ranges for the estimation of global mesopelagic fish biomass. ICES J. Mar. Sci..

[9] Benoit-Bird KJ, Au WWL (2006). Extreme diel horizontal migrations by a tropical nearshore resident micronekton community. Mar. Ecol. Prog. Ser..

[10] Tuya F (2012). Patterns of landscape and assemblage structure along a latitudinal gradient in ocean climate. Mar. Ecol. Prog. Ser..

[11] Kampa EM, Boden BP (1954). Submarine illumination and the twilight movements of a sonic scattering layer. Nature.

[12] Frank T, Widder EA (2002). Effects of a decrease in downwelling irradiance on the daytime vertical distribution patterns of zooplankton and micronekton. Mar. Biol..

[13] Røstad A, Kaartvedt S, Aksnes DL (2016). Light comfort zones of mesopelagic acoustic scattering layers in two contrasting optical environments. Deep Sea Res. Part I.

[14] Aksnes DL (2017). Light penetration structures the deep acoustic scattering layers in the global ocean. Sci. Adv..

[15] Bianchi D, Galbraith ED, Carozza DA, Mislan KAS, Stock CA (2013). Intensification of open-ocean oxygen depletion by vertically migrating animals. Nat. Geosci..

[16] Netburn AN, Koslow JA (2015). Dissolved oxygen as a constraint on daytime deep scattering layer depth in the southern California current ecosystem. Deep Sea Res. Part I.

[17] Klevjer TA (2016). Large scale patterns in vertical distribution and behaviour of mesopelagic scattering layers. Sci. Rep..

[18] Proud R, Cox MJ, Le Guen C, Brierley AS (2018). Fine-scale depth structure of pelagic communities throughout the global ocean based on acoustic sound scattering layers. Mar. Ecol. Prog. Ser..

[19] Sutton TT (2017). A global biogeographic classification of the mesopelagic zone. Deep Sea Res. Part I.

[20] Proud R, Cox MJ, Brierley AS (2017). Biogeography of the global ocean’s mesopelagic zone. Curr. Biol..

[21] García-Seoane E, Wienerroither R, Mork KA, Underwood MJ, Melle W (2021). Biogeographical patterns of meso- and bathypelagic fish along a Northeastern Atlantic transect. ICES J. Mar. Sci..

[22] Magnússon J (1996). The deep scattering layers in the Irminger Sea. J. Fish Biol..

[23] Peña M (2014). Acoustic detection of mesopelagic fishes in scattering layers of the Balearic Sea (western Mediterranean). Can. J. Fish. Aquat. Sci..

[24] Ariza A (2016). Vertical distribution, composition and migratory patterns of acoustic scattering layers in the Canary Islands. J. Mar. Syst..

[25] Blanluet A (2019). Characterization of sound scattering layers in the Bay of Biscay using broadband acoustics, nets and video. PLoS ONE.

[26] Cascão I, Domokos R, Lammers MO, Santos RS, Silva MA (2019). Seamount effects on the diel vertical migration and spatial structure of micronekton. Prog. Oceanogr..

[27] Standal D, Grimaldo E (2020). Institutional nuts and bolts for a mesopelagic fishery in Norway. Mar. Policy.

[28] Heino M (2011). Catchability of pelagic trawls for sampling deep-living nekton in the mid-North Atlantic. ICES J. Mar. Sci..

[29] Krafft BA (2010). Distribution and demography of Antarctic krill in the Southeast Atlantic sector of the Southern Ocean during the austral summer 2008. Polar Biol..

[30] Donlon CJ (2012). The operational sea surface temperature and sea ice analysis (OSTIA) system. Remote Sens. Environ..

[31] Castro CG, Pérez FF, Holley SE, Rıos AF (1998). Chemical characterisation and modelling of water masses in the Northeast Atlantic. Prog. Oceanogr..

[32] Machín F, Pelegrí JL (2009). Northward penetration of Antarctic Intermediate Water off Northwest Africa. J. Phys. Oceanogr..

[33] Bashmachnikov I, Nascimento Â, Neves F, Menezes T, Koldunov NV (2015). Distribution of intermediate water masses in the subtropical northeast Atlantic. Ocean Sci..

[34] Valdés L, Déniz-González I (2015). Oceanographic and Biological Features in the Canary Current Large Marine Ecosystem.

[35] Della Penna A, Gaube P (2020). Mesoscale eddies structure mesopelagic communities. Front. Mar. Sci..

[36] Demer, D. A. *et al.* Calibration of acoustic instruments. ICES Cooperative Research Report No. 326, 133pp. 10.25607/OBP-185 (2015).

[37] Knutsen, T. *et al.* MESSOR - A towed underwater vehicle for quantifying and describing the distribution of pelagic organisms and their physical environment. In *2013 MTS/IEEE OCEANS-Bergen.* 1–12. 10.1109/OCEANS-Bergen.2013.6608177 (Bergen, Norway, 2013).

[38] Underwood MJ, García-Seoane E, Klevjer TA, Macaulay GJ, Melle W (2020). An acoustic method to observe the distribution and behaviour of mesopelagic organisms in front of a trawl. Deep Sea Res. Part II Top. Stud. Oceanogr..

[39] Korneliussen, R. J. *et al.* The Large Scale Survey System - LSSS. In *Proceedings of the 29th Scandinavian Symposium on Physical Acoustics, Ustaoset 29 January-1 February 2006.*

[40] MacLennan DN, Fernandes PG, Dalen J (2002). A consistent approach to definitions and symbols in fisheries acoustics. ICES J. Mar. Sci..

[41] Haris K (2021). Sounding out life in the deep using acoustic data from ships of opportunity. Sci. Data.

[42] Geider RJ (1987). Light and temperature dependence of the carbon to chlorophyll a ratio in microalgae and cyanobacteria: Implications for physiology and growth of phytoplankton. New Phytol..

[43] Westberry T, Behrenfeld MJ, Siegel DA, Boss E (2008). Carbon-based primary productivity modeling with vertically resolved photoacclimation. Glob. Biogeochem. Cycles.

[44] Zuur AF, Ieno EN, Elphick CS (2010). A protocol for data exploration to avoid common statistical problems. Methods Ecol. Evol..

[45] Zuur AF, Ieno EN, Walker NJ, Saveliev AA, Smith GM (2009). Mixed Effects Models and Extensions in Ecology with R.

[46] He, S. *Generalized Additive Models for Data with Concurvity: Statistical Issues and a Novel Model Fitting Approach* PhD thesis, University of Pittsburgh 1–51 (2004).

[47] Tweedie, M. C. K., Ghosh, J. K., & Roy, J. Statistics: applications and new directions. In *Proc. Indian Statistical Institute Golden Jubilee International Conference. An index which distinguishes between some important exponential families*, 579–604 (1984).

[48] Stramma L, England M (1999). On the water masses and mean circulation of the South Atlantic Ocean. J. Geophys. Res. Oceans.

[49] Stupnikova AN, Tarakanov RY, Kulagin DN, Vereshchaka AL (2018). Factors maintaining the identity of mesoplankton communities: Cool evidence from the Drake Passage. Hydrobiologia.

[50] Cook AB, Sutton TT, Galbraith JK, Vecchione M (2013). Deep-pelagic (0–3000m) fish assemblage structure over the Mid-Atlantic Ridge in the area of the Charlie-Gibbs Fracture Zone. Deep Sea Res. Part II.

[51] Olivar MP (2017). Mesopelagic fishes across the tropical and equatorial Atlantic: Biogeographical and vertical patterns. Prog. Oceanogr..

[52] Machín F, Hernández-Guerra A, Pelegrí JL (2006). Mass fluxes in the Canary Basin. Prog. Oceanogr..

[53] Carracedo LI, Pardo PC, Flecha S, Pérez FF (2016). On the Mediterranean water composition. J. Phys. Oceanogr..

[54] Dale T, Bagøien E, Melle W, Stein K (1999). Can predator avoidance explain varying overwintering depth of *Calanus* in different oceanic water masses?. Mar. Ecol. Prog. Ser..

[55] Béhagle N (2016). Acoustic micronektonic distribution is structured by macroscale oceanographic processes across 20–50°S latitudes in the South-Western Indian Ocean. Deep Sea Res. Part I.

[56] Kordas RL, Harley CDG, O'Connor MI (2011). Community ecology in a warming world: The influence of temperature on interspecific interactions in marine systems. J. Exp. Mar. Biol. Ecol..

[57] Maas AE, Frazar SL, Outram DM, Seibel BA, Wishner KF (2014). Fine-scale vertical distribution of macroplankton and micronekton in the Eastern Tropical North Pacific in association with an oxygen minimum zone. J. Plankton Res..

[58] Robison BH (2004). Deep pelagic biology. J. Exp. Mar. Biol. Ecol..

[59] Childress JJ, Seibel BA (1998). Life at stable low oxygen levels: Adaptations of animals to oceanic oxygen minimum layers. J. Exp. Biol..

[60] Herring PJ (1998). Across-slope relations between the biological populations, the euphotic zone and the oxygen minimum layer off the coast of Oman during the southwest monsoon (August, 1994). Prog. Oceanogr..

[61] Prince ED, Goodyear CP (2006). Hypoxia-based habitat compression of tropical pelagic fishes. Fish. Oceanogr..

[62] Love RH, Fisher RA, Wilson MA, Nero RW (2004). Unusual swimbladder behavior of fish in the Cariaco Trench. Deep Sea Res. Part I.

[63] Gilly WF, Beman JM, Litvin SY, Robison BH (2013). Oceanographic and biological effects of shoaling of the oxygen minimum zone. Ann. Rev. Mar. Sci..

[64] Wiebe PH, Madin LP, Haury LR, Harbison GR, Philbin LM (1979). Diel vertical migration by *Salpa aspera* and its potential for large-scale particulate organic matter transport to the deep-sea. Mar. Biol..

[65] Agersted MD, Khodabandeloo B, Liu Y, Melle W, Klevjer TA (2021). Application of an unsupervised clustering algorithm on in situ broadband acoustic data to identify different mesopelagic target types. ICES J. Mar. Sci..

[66] Khodabandeloo B, Agersted MD, Klevjer T, Macaulay GJ, Melle W (2021). Estimating target strength and physical characteristics of gas-bearing mesopelagic fish from wideband in situ echoes using a viscous-elastic scattering model. J. Acoust. Soc. Am..

[67] Olivar MP (2012). Vertical distribution, diversity and assemblages of mesopelagic fishes in the western Mediterranean. Deep Sea Res. Part I.

[68] Fennell S, Rose G (2015). Oceanographic influences on deep scattering layers across the North Atlantic. Deep Sea Res. Part I.

[69] Agersted MD (2021). Mass estimates of individual gas-bearing mesopelagic fish from in situ wideband acoustic measurements ground-truthed by biological net sampling. ICES J. Mar. Sci..

[70] Kaartvedt S, Staby A, Aksnes DL (2012). Efficient trawl avoidance by mesopelagic fishes causes large underestimation of their biomass. Mar. Ecol. Prog. Ser..

[71] Vereshchaka A, Abyzova G, Lunina A, Musaeva E (2017). The deep-sea zooplankton of the North, Central, and South Atlantic: Biomass, abundance, diversity. Deep Sea Res. Part II.

[72] Klevjer T (2020). Micronekton biomass distribution, improved estimates across four north Atlantic basins. Deep Sea Res. Part II Top. Stud. Oceanogr..

[73] Godø OR, Patel R, Pedersen G (2009). Diel migration and swimbladder resonance of small fish: Some implications for analyses of multifrequency echo data. ICES J. Mar. Sci..

[74] Davison P, Lara-Lopez A, Anthony Koslow J (2015). Mesopelagic fish biomass in the southern California current ecosystem. Deep Sea Res. Part II Top. Stud. Oceanogr..

[75] McClatchie S, Coombs RF (2005). Low target strength fish in mixed species assemblages: The case of orange roughy. Fish. Res..

[76] Nychka, D., Furrer, R., Paige, J. & Sain, S. fields: Tools for spatial data. R package version 133. https://www.githubcom/dnychka/fieldsRPackage (2021).

[77] Wickham, H. ggplot2: elegant graphics for data analysis. (Springer-Verlag, New York, 2016).

[78] Sarkar, D. Lattice: Multivariate Data Visualization with R. (Springer, New York, 2008).

[79] Wickham H (2007). Reshaping data with the reshape package. J. Stat. Softw..

[80] Wickham H (2011). The Split-Apply-Combine Strategy for Data Analysis. J. Stat. Softw..

[81] Bivand, R. & Lewin-Koh, N. maptools: Tools for Handling Spatial Objects. R package version 11–3. https://www.CRANR-projectorg/package=maptools (2022).

[82] Wood SN (2011). Fast stable restricted maximum likelihood and marginal likelihood estimation of semiparametric generalized linear models. J. R. Stat. Soc. Series B..

[83] Pebesma EJ, Bivand RS (2005). Classes and methods for spatial data: the sp package. R news.

[84] Bivand, R. S., Pebesma, E. J. & Gómez-Rubio, V. Applied spatial data analysis with R. (Springer, New York, 2013).

