# Supplementary material for: Acoustic micronektonic distribution and density is structured by macroscale oceanographic processes across 17–48° N latitudes in the North Atlantic Ocean
Source: Sci Rep. 2023 Mar 21;13:4614. doi: 10.1038/s41598-023-30653-5 (PMC10030644; doi:10.1038/s41598-023-30653-5)

# Supplementary information

**Figures**

**Fig. S1** Vertical profiles of total organism density estimated from the acoustic records from MESSOR in 3 stations at 120 kHz (a) (c) (e) and (b) (d) (f) 200 kHz.

**

**

**Fig. S2** Plot of the Pearson residuals against spatial coordinates for (a) surface micronekton at 18 kHz, (b) micronekton from deep layers at 18 kHz, (c) surface micronekton at 38 kHz, (d) micronekton from deep layers at 38kHz, (e) surface micronekton at 120/200 kHz (f) micronekton from deep layers at 120/200 kHz.

**
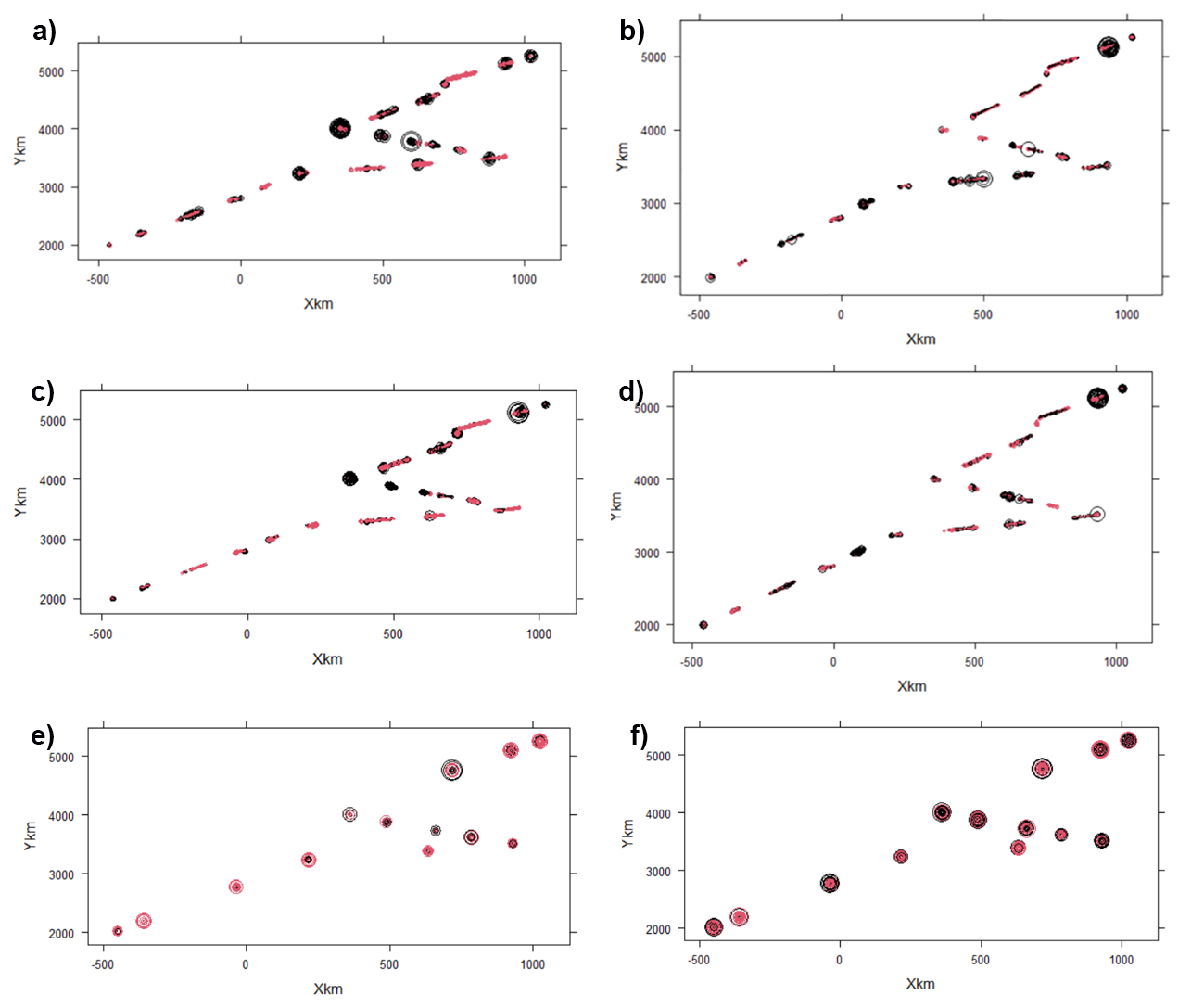
**

**Tables**

**Table S1** Water type characteristics for upper and lower North Atlantic Central Water (NACW), Subtropical Underwater (STUW), Antarctic Intermediate Water (AAIW), and Mediterranean Overflow Water (MOW) (e.g., Bashmachnikov et al. (2015); Castro et al. (1998); O’Connor et al. (2005)).

| **Water type** | **T (°C)** | **S (psu)** |
| --- | --- | --- |
| Upper NACW | 18.0 | 36.5 |
| Lower NACW | 9.5 | 35.3 |
| STUW | 21.0 | 37.0 |
| AAIW | 6.5 | 34.9 |
| MOW | 11.0 | 36.2 |

References

Bashmachnikov I, Nascimento Â, Neves F, Menezes T, Koldunov NV (2015) Distribution of intermediate water masses in the subtropical northeast Atlantic. Ocean Sci 11:803-827.

Castro CG, Pérez FF, Holley SE, Rı́os AF (1998) Chemical characterisation and modelling of water masses in the Northeast Atlantic. Progress in Oceanography 41:249-279.

O’Connor BM, Fine RA, Olson DB (2005) A global comparison of subtropical underwater formation rates. Deep Sea Research Part I: Oceanographic Research Papers 52:1569-1590.

**Table S2** Spike and bubble filters/modules applied on the hull-mounted acoustic data using the pre-processor KORONA in LSSS.

| Type of filter/module | Applied depth range (m) | Total delta (dB) (b) | Vertical delta (dB) (c) | Vertical unit | Vertical median search duration (ms) (d) | Window median search duration (ms) (d) |
| --- | --- | --- | --- | --- | --- | --- |
| Spike filter (a) | 90-250 | 14 | 14 | Duration | 0.9 | 4.4 |
| Spike filter (a) | 240-2500 | 10 | 10 | Duration | 0.9 | 4.4 |
| Spot noise filter (f) | 10-2500 | 14* | - | - | - | - |
| Bubble spike filter (e) | 10-100 | 14 | 14 | Duration | 2 | 4.4 |
| Bubble spike filter (e) | 90-250 | 10 | 10 | Duration | 2 | 4.4 |
| Bubble spike filter (e) | 240-2000 | 7 | 7 | Duration | 2 | 4.4 |

a) A spike filter is usually not needed close to the surface since the spikes there are not amplified by the TVG. Further, a small and strong school may be taken as a spike. For 1 ping/s, 7 degree beams are overlapping at approximately 50 m depth, for 0.5 ping/second at 100 m. Therefore, the first spike filter starts around 100 m

b) Min. difference of current sample to search window median to be spike candidate

c) MIn. difference of search column median to (most) neighbouring pings to be spike candidate

d) Half the height of the search column/window

e) BubbleSpikeFilterModule works in a similar way as the SpikeFilterModule, but looks for measured values with a vertical extent that is much weaker than the surrounding values.

f) The SpotNoiseModule removes a single sample that is much stronger that the surrounding samples. This does not occur often, but some instruments, e.g., the Simrad EK15, may occasionally have this problem.

* Delta: Spot-noise candidate if center value > 93% percentile + Delta

After applying all the above filters, a “Fill Missing Data Module” was used. This filter/module duplicates the previous ping for a frequency if there is a time where there exists data for at least one other frequency.

**Table S3** Summaries of results from the GAM final models for:

1. Micronekton from surface at 18 kHz.
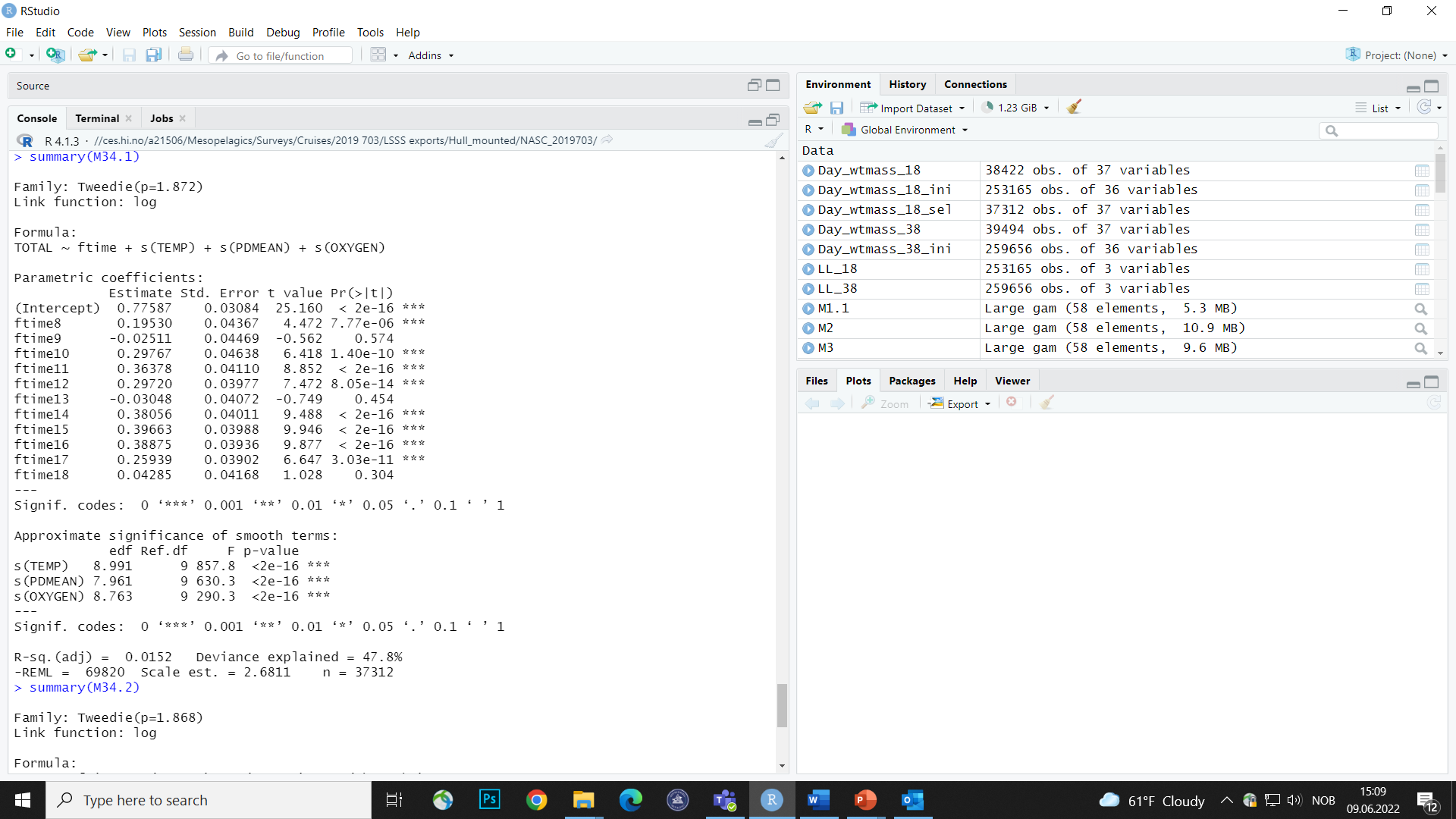

2. Micronekton from surface at 38 kHz.
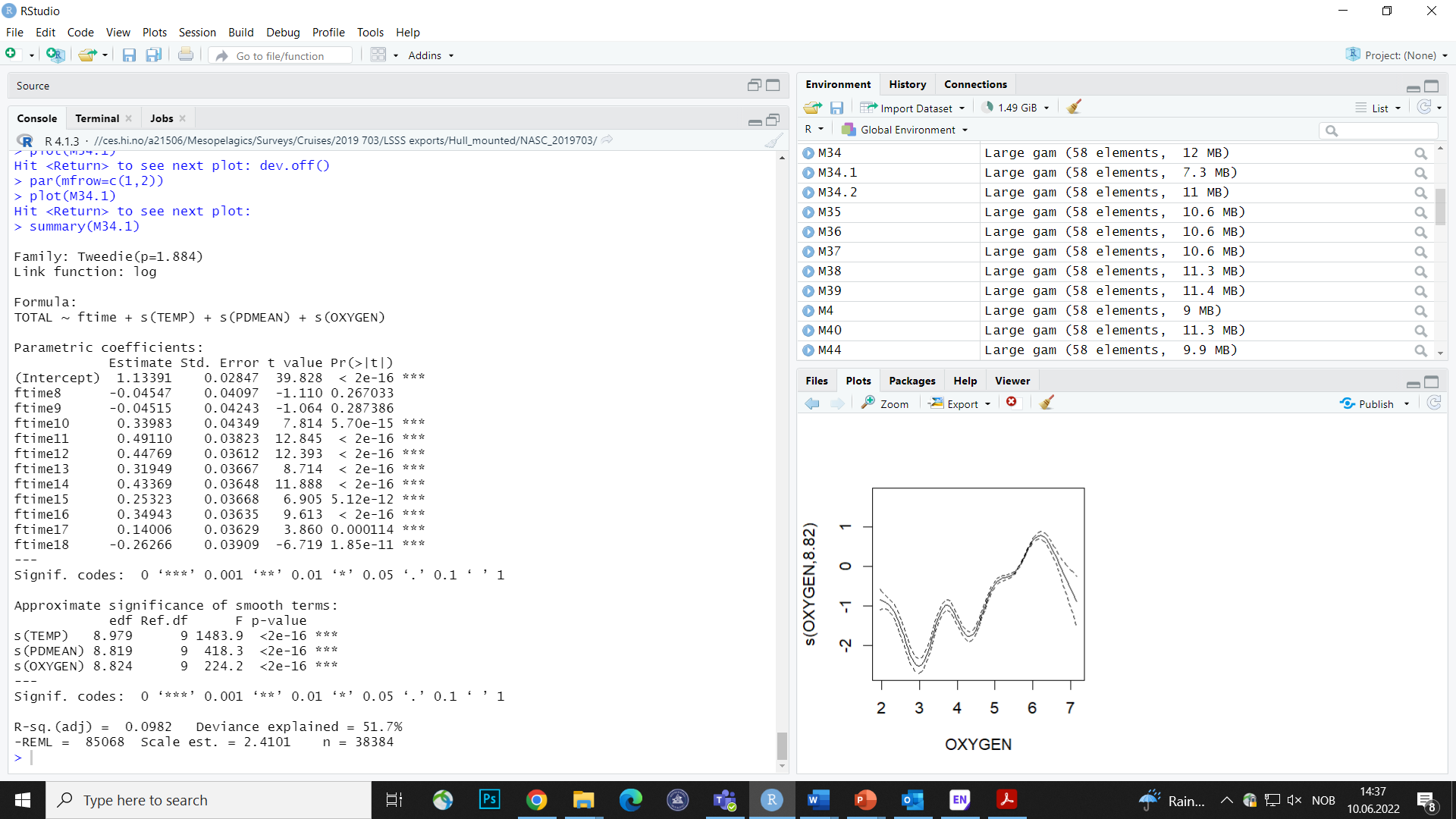

3. Micronekton from surface at high frequency.
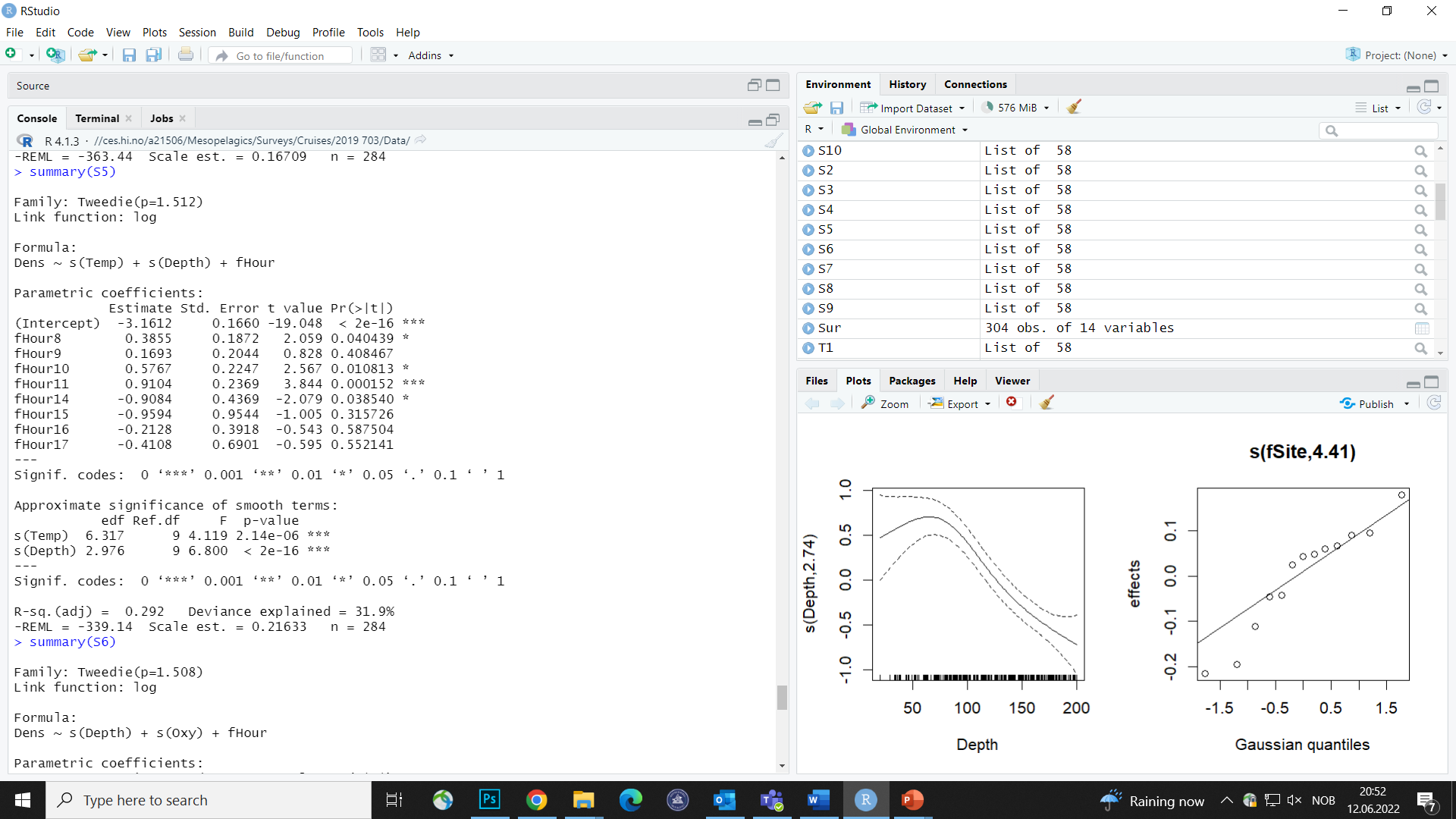

4. Micronekton from deep layers at 18 kHz.
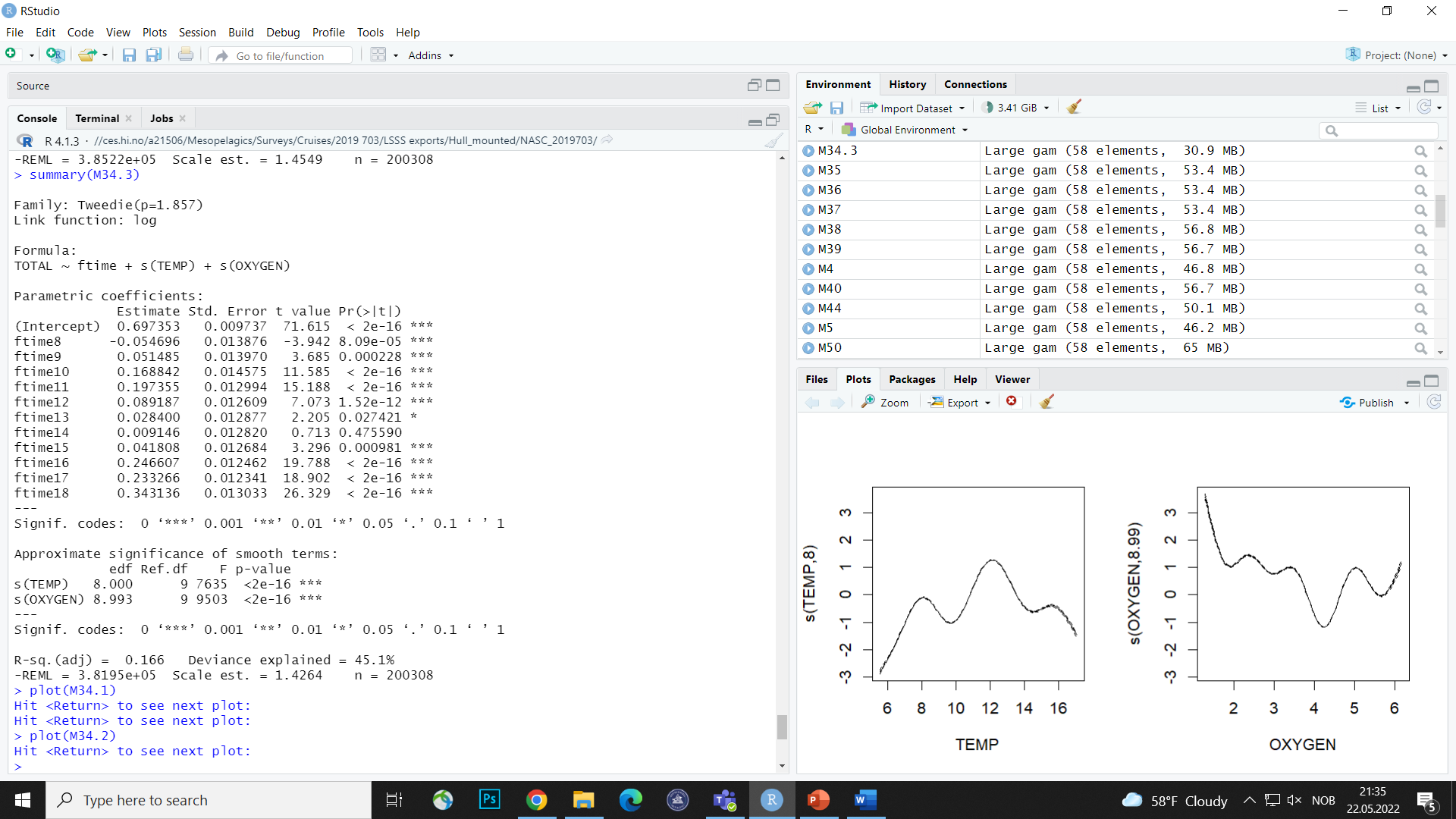

5. Micronekton from deep layers at 38 kHz.
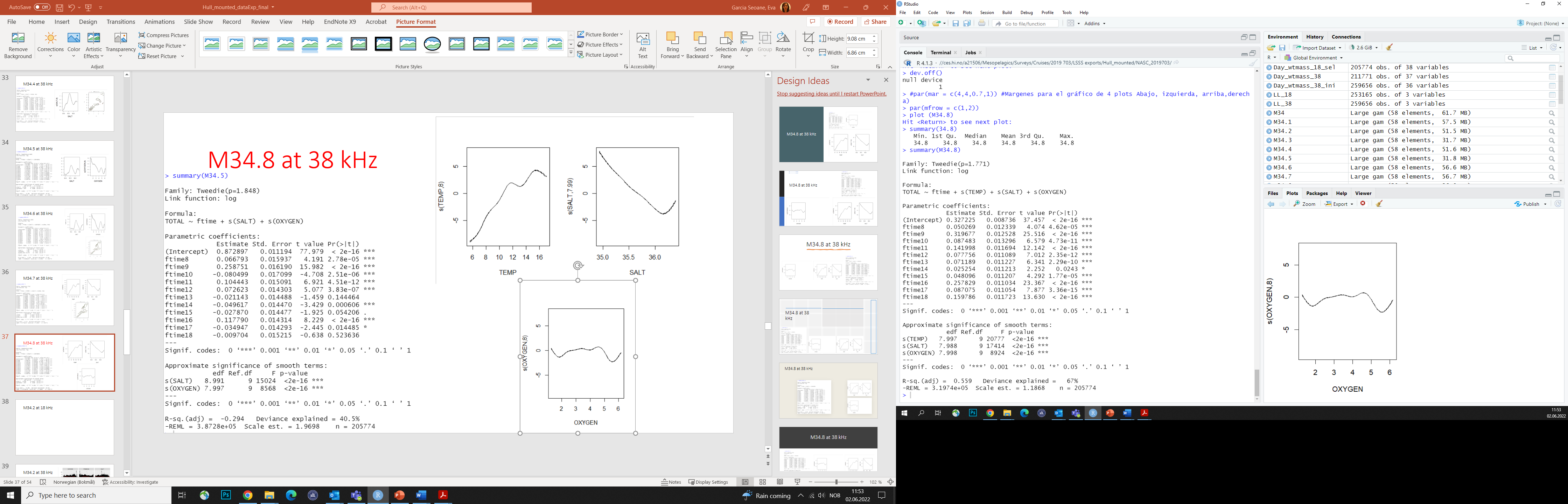

6. Micronekton from deep layers at high frequency.
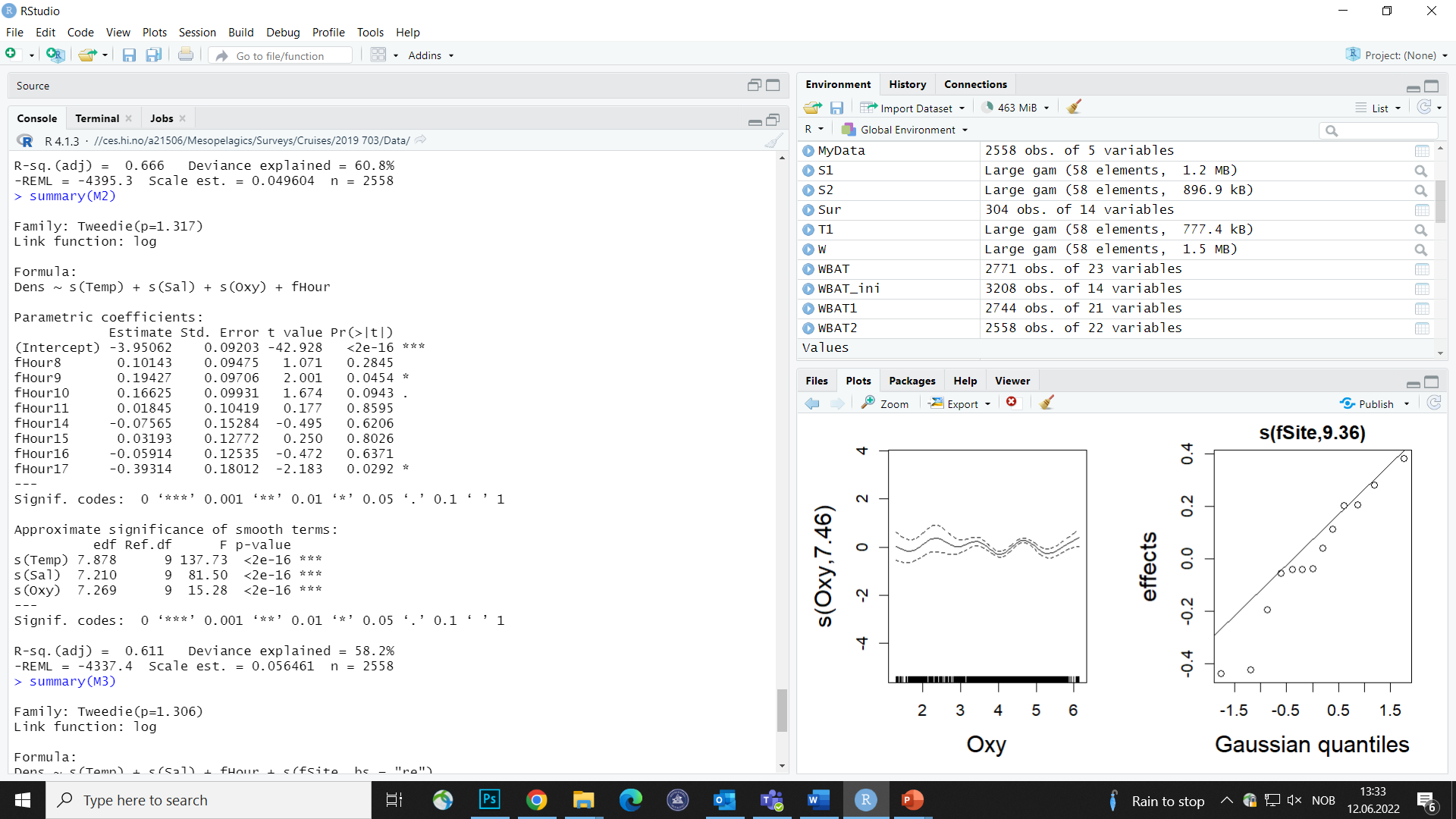

Supplement: Supplementary file 1 — Supplementary Information. [file 41598_2023_30653_MOESM1_ESM.docx]
